# Supplementary material for: Genetic and behavioral analyses suggest that larval and adult stages of Lucilia cuprina employ different sensory systems to detect rotten beef
Source: Parasit Vectors. 2025 Jul 9;18:270. doi: 10.1186/s13071-025-06804-0 (PMC12239484; doi:10.1186/s13071-025-06804-0)
Supplement: Supplementary file 1 — Additional file 1: Fig. S1: larval stages and tissues used for the RNA-Seq experiment. (A) whole larva stage 1 (L1); (B) whole larva stage 2 (L2); (C) lateral view of a whole late larva stage 3 (L3) and (D) ventral view; (E) whole early larval stage 3 (WL); (F) first segment designated as “head” (H) from an early L3, detailed within a dashed white circle; and (G) gut from an early L3, where crop have been separated from the cardia originally connected by the foregut (not present in the picture); Note S1: LcupOrco gene, guide RNA (gRNA) sequence, and primers used to determine potential indels and point mutations at the gRNA cutting site (genotyping); Fig. S2: LcupOrco insert construction. (A) LcupOrco gene fragment including two sequences corresponding to the left (LHA) and right (RHA) homology arms of 1000 bp in length each, necessary for the CRISPR homology directed repair (HDR) protocol cloned using plasmid pUCIDT-AMP GoldenGate (ordered from IDT). In addition, a DNA section including the restriction cutting sites (RCS) for enzymes XhoI (NEB) and NotI (NEB) was added in between of both sequences in the same plasmid; (B) donor plasmid including the ZsGreen Marker followed by the hsp83 promoter located between positions 1891 and 7441 [2]; (C) ZsGreen Marker obtained from the donor plasmid. The plasmid was linearized using restriction enzymes BpmI (NEB), XhoI and NotI at 37 °C for 60 min, and the reaction was finished at 65 °C for 20 min. After restriction digestion, the mix was electrophoresed on a 1.5% agarose gel at 60 V for 120 min. The band with the size corresponding to the marker (~5 kb) was recovered from the gel and purified using the Zymoclean Gel DNA recovery kit (Zymo) following the manufacturer’s specifications; (D) to obtain the final construct, 50 ng of linearized pUCIDT-AMP GoldenGate plasmid + 175.5 ng of the marker fragment (1:3 ratio) was ligated overnight (O/N) at 16 °C using the T4 DNA ligase (NEB). After ligation, 1 μl of the reaction mix was us [file 13071_2025_6804_MOESM1_ESM.pdf]

Genetic and behavioral analyses suggest that larval and adult stages of *Lucilia cuprina* employ different sensory systems to detect rotten beef

Juan P. Wulff [a], Rachel K. Laminack [a] and Maxwell J. Scott [a]

Supplementary Methods

Figure S1: larval stages and tissues used for the RNA-Seq experiment. (A) whole larva stage 1 (L1); **(B)** whole larva stage 2 (L2); **(C)** lateral view of a whole late larva stage 3 (L3) and **(D)** ventral view; **(E)** whole early larval stage 3 (WL); **(F)** first segment designated as “head” (H) from an early L3, detailed within a dashed white circle; and **(G)** gut from an early L3, where crop have been separated from the cardia originally connected by the foregut (not present in the picture).

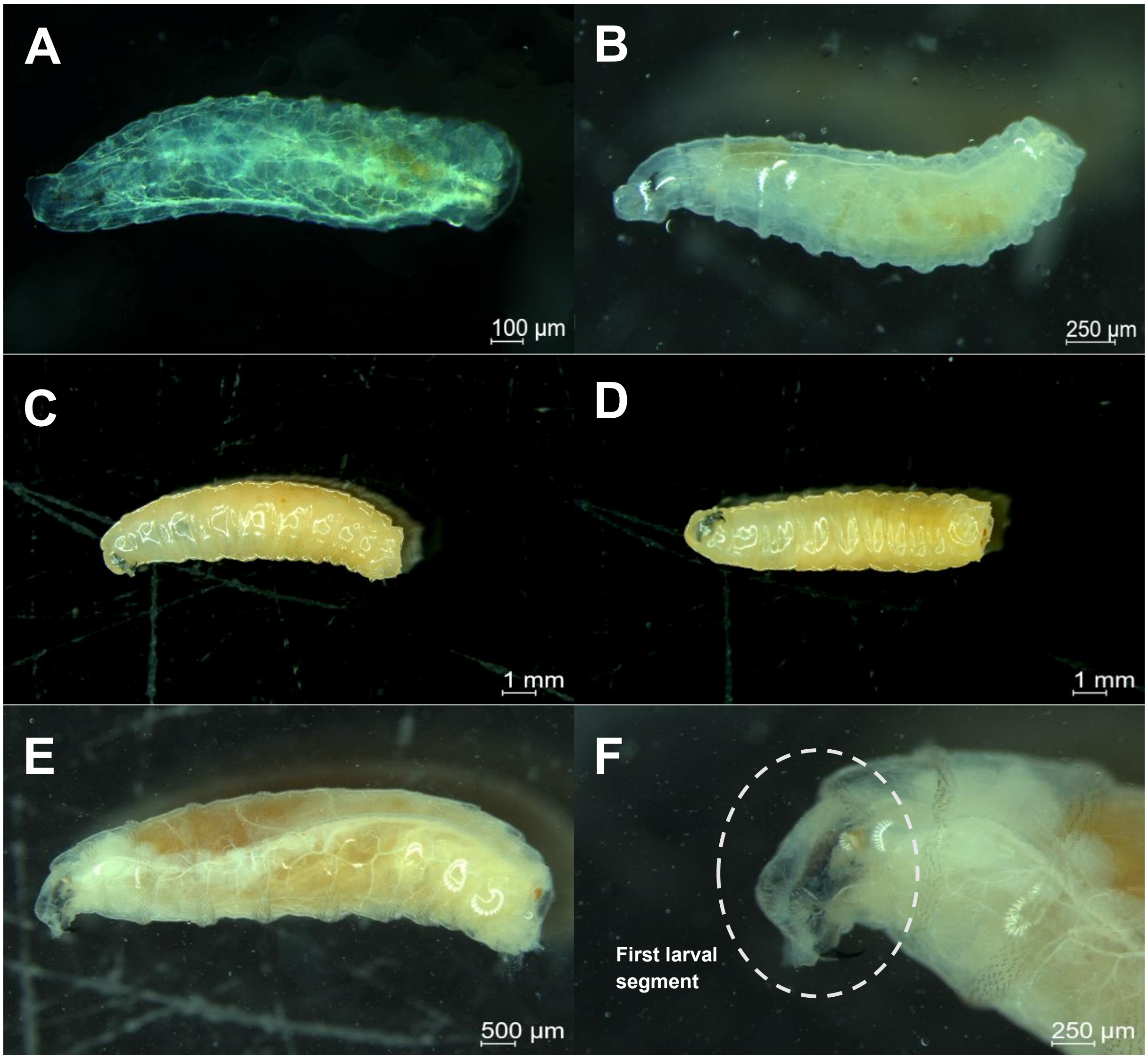

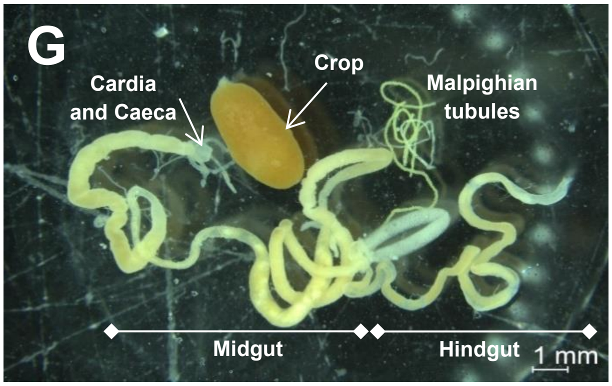

Note S1: *LcupOrco* gene, guide RNA (gRNA) sequence, and primers used to determine potential indels and point mutations at the gRNA cutting site (genotyping).

(A) Orco gRNA (Lcup-Orco-crRNA1) without the PAM sequence at the 5'-end. The ortholog sequence of this gRNA was previously used to edit the first exon of the *LcupOrco* gene in a species closely related to the fly under study, the new world screwworm *C. hominivorax* [1]. Its location, including the PAM sequence underlined, was highlighted in bold in the *LcupOrco* gene sequence provided below.

Lcup-Orco-crRNA1 sequence: GACGAAGTAAATGAGTTGTC (+)

(B) Primers used for genotyping and to test the gRNA cutting efficiency *in vitro* using the EnGen Spy Cas9. Primers positions were highlighted in bold in the *LcupOrco* gene sequence provided in D (see below).

Lcup-Orco-sgRNA_1_Fw sequence: *ATGCAGTCGAATCTACAACC*
Lcup-Orco-sgRNA_1_Rv: *gccaacaataatcatggtttca*

(C) PCR cycling parameters

Initial denaturalization = 3 min at 98°C.

Denaturalization = 30 sec at 98°C.
Annealing = 30 sec at 60°C. Repeat x 40 cycles
Extension = 30 sec at 72°C.

Final extension = 5 min at 72°C.
Hold = ∞ at 10°C.

The Q5 High-Fidelity Taq Polymerase 2X Mix [New England Biolabs (NEB), Ipswich, MA, USA, Cat. # M0491S] was used for DNA amplification. Reaction was performed in 20 µl of final volume and 300 nM for final primer concentration.

(D) *Lucilia cuprina Orco* gene sequence including 5'- and 3'-UTR regions, detailing exons and introns as UPPERCASE and lowercase letters, respectively, primers positions and gRNA highlighted in bold and PAM sequence underlined.

```
cttaatatttaaagtttatttttaatttttttacaaaagtcgaaaatttaaaaggtatgcaaagtagggagaaaactgaaaaagttaatacttaaagtatgtacaaattataa
gtacattatacatggaaaattatgttaaagtgtatttaaaccttaatcattacaggcgaaaccgaagtgtattttccaaaaaggattatattgattaaagcaatatttaaaa
aatatttgtttgaattcagaaatatctatttttatactctacaccactataattaggagtgtattatgtgttggttctgacatttgtaaatattggtccttatatatatcag
tcagctcagcatcactttctgagttgattgtggtatgtctgtctatgaatttcgacacatactagtttctttatccaaggacatttgacttatgtaaaatctggctttattt
ggatcatttggaccgattgtggtagggtcaattagggaccggttcacagtggtatattaaaaagagggaaataattcggtaacttctaaacggtaaatccgattttaatgaa
atttggtatgcacaaagagaaggtgttgtcgagcttaggttttgaatttggaccttaaaggccaaccagggacccgatggggggtcctcaaattaggacacctcggctatgt
taaattttaaaatgatcctatttcttcttttgagttccgatttaaaaaaaaaattcgtttatagaatctcctcatcaaacactataaaaaatgtcgtagcagtaaattatc
ttttatagttgaggatatattctcatttgaaaatcaaaattttaaaatttttaccgtccttactttagtttttttgataatagcgggtcagaaaaattcccgattttcgccat
ttttattcgcttaacaacaagtttatatatcatgcagtgaaaaaaattatgtaaaaatcatgactgagtccaaagttataggcattttaatttaataaattaaaaaaggcga
ttttttgccatttttttgggaaaaagtatctttttctttttaagttatctgaaaagtttctaataagatgtatatagaatttatactttttttgaaagctgactttacataaat
acgttaaatgaaacaaaacctaaaactttttgttaccgagtggaccaggtccatccaaaaaaaaccctattttttaatgaaaattcaattttgagcaaaaattctcaaatcg
catagtcgatatctaattatagtgacctttttttatatgacccaatatgttcttaatcatttgtaacgggtttcgataaccccgccactggtatggatgtaataggcaaaaa
accaaaaaatccaattttttgggatttttttaaactttttttgggaattccgggatttctaataagaaaattcgaaattttttatttaattttatatttgagtaaaaaaatctac
caaactgtaaaattttattaaaaaatattcataaataaaattttttattgctatttgaaaaatttgtatcttcgcaataactgaataaaaaacatttttttaattttttttaaa
aagtattccgcgatttttttaattttaaaaattatatacagttttgaaaagtagacaaaattatataacatacctacctaatgtttttaagtgacaaattaattagggaaaa
ctcaacatcttttagaaaatttacctagtgctcgatgaggagattctatatacgaaatttttttaaatcggaactcaaataaagaaataggatcgttttaaaaatttaacat
agccgaggtgtcctaatttgagaacctgtactcgttcctgtactcgtatttctccacagtgataaaagtttaagtcacactctgaagtagatcttatagaagggattgtgcc
aattatagaccgatcctaataaaatttctttaatagatttaagttcatataagacttgtttatggagaattttagctatgactgttgtaattgttttcccaagttccactta
tattatccatatgcgtaaaattttaactctatatatctttaatcattcgaaccctatcgtgctttaaacagacagacggacatggctagatcatcttacaatttaatgagga
```

cccagaatctttttttgatggtagttataaaaaatctgctgtaggtccgattgcgtcctcaagacaagtctgaggaagatataatttcaccaaaacgaagtctattgctacaa
atttttctgtccgtccgtccgtccgtccgtccgtccgtctggctgtccatgtaaaccttgtgcgcaaggtaccgcaattttcaagataattggatgaaatttggcacaaaca
ccttttaggcccaaggacgaagcctcttgaaaatggttaatatcggtccattatttcgactatcccccatacaaccgtaccccccaaatagggcctttgggcttataatta
atttaaatgatctattatgtcaacaaaagtcgagaaaactaagttttatagaactttaaatgacactaccgattttttgtaatgattgggctacatttgaccctagcccccat
acaaactccccttcagaaaatgacttaaaggtcaaaattcacttataaacactaatgacacttttaaattctacataaataatattgaagtagacttaactcccccctaccaa
catttataaggatagggccatatttgccctactcccctttgagccctcttgtaaaaaatttatttttggcaaataaaaaataaataaaaaatattccgaaataaagttaaaaa
caaatcaaatgctttatttttttaaataatccccattttaacatacatactgactggtgtagggtatcatatggttggctacatccgactatacattcatacttgttttaaa
ttataaacatagttaagacttattgaagacttcgcttaatataagtctaaactatgttttaagtatttttgtagaaataagtactagaacaattaacataaatttagcctga
aaagccatatttgttaggtatggttgtatggaggccatatattttttcttttttttttcattacaaacataagcgcaaacccaatataacttcccttagtaaagtggtgtagg
ctaaacttaggtcaacttgaatcttacagattcaagtttggaagatttcctaaaaaaagaaatacttttgctaaatttttgaaaatataatttgacttaattctttaatgtt
tatattaaacctttttctaaatatatcatatttcagataataagagagatatgtctgaagtttaaataaaatcaaataatgttaacccataaactgtgaagctgaaaatcaag
ttatgcctatttttatgaaaccttcactattatttaattttaaaccgatttaaaaaaaaaaaaacttgagtaaagtatataataccgcaggaataatttcagaaattgttcta
aaaacaactagttttagaatgaatgcaaatagaatcactttagattctttaacaacaaccttttcactaatgtgtcgtcattatttgtaaaaccctttcttcggaacattgt
tatggttcttaaagagttttaaggaactagttctttgatactaattatcagggctaggatttctatgcaaatgcatatttttagtcagtaactcaaaataacttttaactag
ttttcttttcaaatcaaagaattttctacaaaatggcatcgatttgttgttgcatattttgttataaatacataaattgcatattttgctttaaattgcatatattttgcat
attttttaacattttttattgcatattttgcatgtttttttaaaatttttctaaaacaaattgttttgttttttttttctgttttttatattttttaaaacaaattaggtatttat
gtacatatttagatttttttaaataactttcaacaaatttcaatttaaaaaaaaaaatactaggttctatgaaatacaatttacaatcacgtccaaagtattggtccttaaag
ttcagttcaatttctgagtctgtccatatgagtgtatatataaaaaggtaactagatgaaattttgttcttagatctctctcacgttcataattcacttaaaaacattacca
aacgataaaaattcggaagaaatgtaatttatacaaaaatatgaaaattcaccaaattttattagaattgccatcaagctttccgcttacagaaattagatatatgtatacta
ttttaagttgtttcttagtctttaggttagtggtcagtgttctagtctggtagaccggagttggtgggttcgattcccacccgtggcactggttaaggagcgcacaacaggc
ctaggtgtatttcttcgaatttgatgtgtatacttacatcctcctttcaaacttactaactaactaacttagttaactacctaactaacttactagtctttatatatgtcat
tctcgggcttgtggttccgttaaagtggtattatagtacataaaacagtccttaataacctgtaaaaacttaagacctgattatatattaatatgtactttcgcagagaaat
attttttggtacattttttttcacattttttagcgcatatttaccatttttatactgtataaaacataaattttaaatttttagtgcatgaaaatcctttgcttgctaaagagt
tttaagaaactagttctttgatactaattgtacattgattctatactagtttaatggaatgcgtttctcaaaattgattagcgaatacattctccaaagatttcctagaact
agttccggacttgacaaattcgaactaaaatgattgatttaagaactagttccagaaccgttccaaagaatgagtgctgaatccatagcgcttcctcagaatcagttacgat
gctgacaatttagaacaacaataattcatctcaatgtcaaagtagaaagagtacaggctttaatcacaaaaactgacaaaatgggcggaattactatagtgggcgcggcatc
ttccatattaagaaaatattaaatctgtttatctcagaaaatgtaacagagccatcacaagtttcaatgtgattagttaggataaaaaatgggcagactagcagcagtgggc
gtggcaactcccagaaactaatagaactagattactaaaattttgcacaattgactttcatattcaactcaacattttgggaaaataaccggacgttgatcaggtggtggct
tggaacccccatatgaattacatgggttgattggcccctcccatatgaattgtatacaatacaattttatttaatttttttcatagcttacattaaaatcctaattagggttc
gataaaattagcgaacttgcaataatttgagtggtatctcccatgagtaagtgggtttttttgcctttaggatagcaaagcatactgggtatatctagtattataattatat
aaaatttccgtaatatatgtaccaaatatttattctatattagttcttaaagtgcttaccaaaatttatttcgttgacctgtgttattccgataccgaatcatttgctcgt
gaaataatataaaattatatataaaaatgacagcttgttgctttgatcgaaattattggatcaatcgtccattaagataaatatttttgtattgaaaaaaaatattaaatgtat
ttttcttaattgatccctttgaaattttaacaattgcattatctcactttaccttatctccatgcag***ATGCAGTCGAATCTACAACC***AACGAAATATGTTGGCCTTGTGGCC
GACTTAATGCCCAACATTAAGCTGATGAAATATTCTGGTTTATTTATGCATGCGTTTACGGGTGGATCACCGCTGTTGAAAAAAGTCTATTCGTCCATACATTTAGTGTTAA
TATTGGCACAATTCATGTTTATACTTGTCAATATGGCTCTGAATGCT**GACGAAGTAAATGAGTTGTCCGG**TAATACAATTACCGCACTATTCTTTACGCACTGCGTTACGAA
ATTTATTTATCTGGCTGTGAATCAGAAGAATTTTTTACAGgtaagtgatgggggtcaccactaaacggcggagtatggatgtgaggattgtttgtatgtgtgcgtgtgtatgt
gagtgtttgtttaatgggcgactatgggattgaaaa***tgaaaccatgattattgttggc***ttttaattgctacttgtgcaaaatgcgaacaaaatacaaggaataaattaaatc
tggcctctgacatggttgacaatattaaatgggtcttttttttattgtttgcaaactgtgtctgtgctagtgactaggctgagctgtgtgccacagtaaaaatgtcacaagaa
tcaattaatcgagtaatttattttgcattaaaagttattctttgttctttctttttcaaccattaactttgtggtgtatctgcagAACTTTGAACATTTGGAATCAGGTTAA
TACTCATCCATTGTTTGCTGAATCCGATGCTCGCTATCATTCAATCGCTTTAGCAAAAATGAGAAAATTATTCTTCTTGGTAATGCTGACGACAGTAGCATCTGCAGTGggt
aagttaaatgcttgggaggagtttatgcctgaacgtgtaatactgtgattaaagtggtaattaattaaatttatctttgtactttaGCTTGGATAACCATCACTTTCTTTGG
TGAAAGTGTAAAGTTTGCCTTCGACAAGGAGACCAACTCCTCTATAACAGTGGAAATACCCAGATTGCCTATTAAATCATTCTACCCCTGGGATGCTAGCCAGGGTATATTC
TATACGATATCATTTGCCTTTCAAGGCTACTACTTACTATTCTCCATGGTACACTCGAACTTGTGTGATGTGCTCTTCTGTTCGTGGTTAATATTCGCCTGCGAACAACTGC
AACATCTAAAGGGCATTATGAAACCTTTAATGGAATTATCTGCCTCCTTAGATACATATCGTCCCAATTCAGCAGCACTCTTTCGTTCACTTTCTGCCAACTCCAAGTCAGA
GTTAATCATTAATGAAggtaagccaaaagacagctgccagccagagtgttttcagttgatgatgtatgtatgtgcataaatcaattttatttttaatttcgttttataGAAAA
AGAACCTCCCAGTGACCTGGATATGACCGGCATCTATAGTACCAAGGCCGATTGGGGTGCACAGTTTCGTGCGCCCACAACACTACAAACATTCAATGGTGTCAACGGTGGC
AATCCGAATGGTTTGACAAAGAAACAGGAAATGATGGTACGCAGCGCTATCAAATATTGGGTGGAGAGGCATAAACATGTTGTCAGgtaaataagttagcgacccaactaac
tccatccattctaactaagtagcataaaaattcgtttattgaacccaagtttgtgtcagagttctttatatttgttcaatcgtaacgaaaaggaaaacataaacaaaacgtc
agtgatggaaggaaggaaacagttgttgtttaaggaaatttaaattttaattacaaacgaactgtgtaatgtcctttaagcatagaaaacactcagagaaatgtgttagtta
atgaacagaaactagtatatatatcggcttcgggttcttaatattgatctcctcctgagacatccgtgtaaagaatattctgcttccggtaaaggatcgaacacgtctgtcc
aatatgttaaatatatattatcaacgtgtccttaaaccggatttgatcaaaataatgttttgataaagataagaacatgcagactgtggtttcgacattatatcttacttaa
ttttcggtcagaggaatctttcaaatagtaccacatacagccacgtgcccagtttcttaaattggaattggtccaactataatttttaagtcaatatttcataattgaatta
agactctattttatctgttatattaaacagatcggataacttgttagtgttatattttgatatttccattttagtttctgattaaaccagtggtgctcaaacacatactatc
atagatagttttatgtgtgttggtaaggcagcagagaacagaattgcattcaaaataaaagtatactaataaaatgactccaaactataaagttaatgctaactgttaaata
tttattatggattttgacagcgagcgtgtatcaaagtctatatataagaaactatcaactgttctctcatgttctactacattaattcggctttcgttgacaaatgttgatc
agcaactgatttgattttgtttgagagcaaaccaactgatattataaatagaggaagaggggccaggtgaaataatagaccgatcttaaccattttaataggctttgtccc
aggatattgaatagaatattgtgtaccaaatttcattgaaatatctccaaaatttcgacctgtagtttgattaaaaggtttgcatggacggacaggcggacggacatagcta
aatcgactcagaaaatgattctggtatactttaaggtggatatatacgaccaatattattgtgcgttataaattgttatagctagaatcttcaaaaactttgggtagcaaa
gcacacagggtatagctagtatacatatatattcagggaatatttttataaatatattgtagtacaattccttgaattagagccccttgcagatcatcttaattaaataata
atgtttcacacataacaaaattcaatttctaatttttttgaaacaatttttactgtcgattttctgtcagtgtaactaatgctgaagtattaaagacaataaacaaggccct
ttcgtttttttcagtctattgcaatcgaataaagttttttttataagtaacttagtttgtttgttaaactcatcactttttatttactatttatagacaaatggtttgtttagg
ttttcaacagttatagatccttctattgacgcgtccgtccagctgtccgttttctatttgtctattccttcttttaattttgttgcatgtttatttgtggcaaaaaaaaaaag
taaaaccaatggaaaattcacagataaaaaataaagccaataagcattttctatatctaacatattcatagtcctgactaaatccatcatcgtccatcatgtttgcatcgat
gatttggcttttattatcctatgtttgctggagtgtgtctgtacatctgactgtttggtttccgtttccatgtttgtttaatgtctgtttatgtatatgtatagttgggtat
taattgaaataaaagtaaatattttaattccttaaagtaacattgctgaatatggtatttagtcttattgatttttctctgatttctccattaatttatttatatattttta
tttctttgagttttttattaacacattattttatctaaactgttgaactaagaggattatttttcatatttattagacaaattaaatgaaattaatatttaatgaactagact
agtggtaaataatgatttttttaagaaatctattaatgtgcttggtttatagtacagacaaaagcaaatgttaatccagggactattgtacacttgactaaaggacttacca
gcgctgaatctggcctctaagccatcgtgtcaggaagtttcaaaaaacacctttttaatcaattttaagacgctataacagtggtcagttatgaatttttatgccataatta
cgttaatatccctctacctttccaaaactttgcattttttttaatgataagaaatgtcttaaacgtccaaaattagtatcgtaatgaaattctgcataagaatttgtaataca
aaatacatgtatatcctttccctaaatttaacaagtaagagtgatatattcgaatataccgaatcaaaccgtttgcagtaaatgtaaaacgttaggttgatattattatgag
attttggcttgtacaaaacatacttctttgtatcgaacttcatagctatactagcatttttttagccagtaatgttcgtttaagccaatttctaaaggggggccttatatgggg
gcagggtcaaccatggaccgattctcattaaattcggtagacagatttgtaattataaggaacttacttgtgtcgaacttcaccgctatactcgtatttttgaagcaagaagt
gagcgtttaagctattctctgaggagaaacttatacgatgagttggataattatgaacggatcctcataaatttcggtagattgattaagctatgtccgtctgtgtgtttgt
tgaaatcagtttttagagtaccccagatatcggcagatacgaatcttcaataattctgttagacatgctttcgacaagatcgcgatttaaaatcagcaaaatcggtccataa
ataccggatatatgagcaaaaagccgagacaacctctaaaaatttcatcaaaaattgagatttttttttcaggctcagacagaaattgcaaaaaacaaaatacataatcatac
ggaaaattttattattgtactcttgtttataaaaacaaggcagagcagcactagtgtgttgttattggctagaaacatgaaaatatgtatgtgaagcctagcttaagttaga
agccataaaagggaacttttttagtacttttttcgttttcaaaaggtactttttgaatttccgatactattgtacctacaaatatgaaattaagtatgtagatgaaaacccata
aaagggatcttttgggtacttttttcgcttttcaaaaggtactcttttgaattttctataatattgaatctagaaacatgaatttgagcatgttaagcccaatgaaagtgatg
gaaaagattactttatggtactgacagtttttgtaacacatcaaggtgaaggttatataagattcggtacagccgaatatagaaccctcacttgtttagtctctagcatttt

ttttttcgaaatatttacttcaacaaaatttgtgttatctatttatcttctccacaaaatgcttcattgatttttttttaaaaacaaggacttgactttttttcaactaaag
ttcacaaataaattctcaaacacatttcttaaatttaaagaatacaaagctcgatgaaatttcatcaccatttcaaataacattatgattataatttgtaaaatttataaat
taaaacaatccccccctagtgtacaagaaagggtattaaagatacgttaaggaaataaagcactttactttcatattttaggttcattattatagaaaattcatcaaatgaa
aacttgaggaacttgatattgaaaattgtgtataatacattcttaggaactgttgcgaaatataaatgtgtttgtttatttacttcgttttttttttttttttttttttgtc
aagtctaaaacacaattactatgtagtagaaaaatgtcaaactaaaagcaaatattgatttatgtaaaaggaatcgaaatgaaatatttcaatatttgtcaaatttatttat
agttttacacacatacctatgtatatttaaattaataaacaaacatttgcacatacacaattgtaaaccttaataaaattgcttttaacaaggactaaacaaactttctata
aatattgtgtaaatgtagatgcttttaacagctacaactacaagtacatgtatttatatttagtacaacaaaaaagaaataatttttaggggaaaaatttaacatgattttt
gtttttcacaaacctacatgccaccctcttccgaccactaataacattgcgttgttgtttttttctcgtgcatttacagATTAGTTGCTGCCATCGGTGACACGTATGGCGCC
GCATTACTTTTACACATGTTGACATCAACCATTAAGTTGACATTGTTGGCATATCAGGCCACCAAAATAACGGGTGTCAATGTTTACGCTTTTACCGTTATTGGTTATTTGG
GTTATGCATTGGCTCAAGTATTTCACTTTTGTATTTTTGGTAATCGTTTAATTGAAGAGgtaagaggtcgattttaaatactattaaatacacacacatctaaatgtactta
aatttgaatacagtatcttgtcggttggttgaagtacattaaatactagttgtattactgctaagcaatatttgtcatagataactacaaagagatgtacattgggtagatt
atgtgtactttgtgctaaacggaagaaattaatttaaacaaacgactaataataaccaataattatgaaataattttaaaatatgtggcaaagctgtagtgtaaattccaga
caaaatcagattatatttagactatatttattccactagtctatagtctagtctatagtctagtctatagtctagtctatagtctagtctagtctatagtcaagtctatag
tctagtctgtagtctagtctatagtctagtctatagtctagtctatagtctagtctatagtctagtctatagtctagtctatagtctagtctatagtctagtctatagtcta
gtctatagtctagtctactgtctagtctatagtctagtctatagtataatatatagtatagtctaaagtctagtctataaactagtctatagtcttgtctatagtgttgtct
aagttttgtctataaaccagcctgttatctaatctgtagtctagtctatacgtaatctacaatcagggctacgaacttgtccataatctagtctatagtctgtattatagtg
cactctgtggtttagaatatagtccatgtactttatggattgtactctataacttaggatctcatttctactatattcgggcctaaaatctggatatatttagtctagtct
gttctatgacaaagttccatgtttagcttacgtctttgacctttacttaacattcaaaatttatgaaaataatataaatttccaaaacatcatattgtgcatgactttaaag
tgtaaatatttattatatttatttagttttgcgttttgtagagcaataaggaaatgaaaaagtagaaaaaaactagactcatgcaagcacaaaaacatttttgaagaacttccg
tttgaaatataacaaaaatagatttttcgccttttcgccactttttcacttttatgaaaaagtttttttgtataaagaaatacacacaggcaacaatgaccatgaaaataaaa
gcacagttaaagaagcagcaaaatatcagcagcacattcactcatcggaaattcgaaagccggaaatgcactaaagtgcttttgttgttttgcgatggtttgaaaatgtata
agtgtgtaagtcttgcacgtatttgtgaatgtgtagaaatttatctctatgtgttagatatttatagaagtgtttatgtgcggctttgtagtctatttatgaacatatatgg
tgtaagtgtatatttgttagcaaatgttttattgttaaagtaaaagttttaaaacttttatttttttacattgctttccaatttaatatttcactattcttatttttcttta
ttttttgtttttcttgccagAGTTCATCCGTTATGGAGGCTGCATACTCGTGTCACTGGTACGATGGTTCTGAGGAGGCAAAAACCTTTGTACAAATTGTTTGTCAACAATG
CCAGAAAGCAATGTCCATTTCGGGTGCAAAATTCTTTACAGTCTCATTGGATTTATTTGCTTCGgtatgttgtttattgtatttttaaattaatttttttctgtgttattta
caaagagacattacaggatatgtgaacaaatatgtacatagagaataacatacaacaaataaacctacacatatcctgacgtttacacaaaggcccacatatcacagacaat
tgattcatacctgtgtgttgtaggtcacacaggaacatttaaaaaaaaggaaacagtgacttgcaaagcaaatatttgccattaactaatattgttatcacaacgtatatct
tacatatacatatgtatatacatatgtatatagatacttcgatttatatttttgaataatatttcaatttttttcaactcagcatggatgatactgtctggtgtgtttgtg
acagttacatttattgtcgcacacaaatgctaaacgtacaaaaggccagacactctagggatcgtatttcaaagggcttttattcgaatttttaacaaaaagtacaaacagg
ctgtaaagtgaatgtcctcaaattgtttttataacattaaaagtgctgttaaaataaaaatggtcatcgttttgttttattcctatagtgaattcaaatttgattgtatagg
ctaagagacctttatacacccagttatgtaacccaagaagcaaatatagctgcattcgtgatcgaaaagatttcttagcacgaatatgtatacgttctttgaacagacaaaa
cgcgacggagggacaatcatacctaacagcagctcgtcacccgcaggttttcaccaaatgcacagcacctccacaccattaaccgccattgccactcagctctcacaagatt
aaattagcaagggccctgtaaaagaacggtcttagtgactcttgacctatcgaaggccttcgactgtctcgccacactcttcgaagatatcctgcaatccacaatgcataac
aagtcgaagaggtggatcgtgaattatataagtggttgtcagtcgtttgtagagttgagagacagacgcttcaaactccgtagagttataccagtcgtactaactaagcact
aacggcacttttgtgcaatttttacctctcgaagctcctcgcacctccacaaggcgtggacgtgatttcatatgcggtcgactgtacgattatggctacggaccacaatatt
aatgaattatgcattgtggtgaatggctatctccatgaaatacatcagttcttcactgcgtcactgggagtgacatttaatagtcttttacttcctcagctcacgtcactg
aaatcacgcacaaattgcgaaaccatgttagctatatataaaacgatcgttcggtctgtggttaattatgctgttccagagtggtcaccttcgctcagtgatacccagtg
gagaaatattcaaagctgtaaaaatgccaccctaaggactgcatacaaattaccttggaacatcatctgtatgaggaaacggaggccctcccagtcaaggaacacaatgtca
tgctgattaaacagttccttctaggatgccatcggaggagtcatcccaacattaacatcatagatggagggacggagggatgctcctccaaggcatgtcgagggaaatatat
gaagatatgcgcaggatcgattggattagactcgaaaacagcagctttgaacgacattcatggagacgccataaatttcgctctcctaggatgccgtatgaatgttgtactt
ggaggtcgcccaccgcctatagcagacacggaaggaaacctgccgcggaaaacaaaagtagttctggcacaactacgatcgggatggagctacaggctcagtgcctactggt
cacgtatagaccgggccgttcaaaacgaatgtactgcagttagagtccccatgacacccacgacatattaaactgcccatccaatcctacttcgctccttccaattgtcaat
catgcagctccaatgtggttcctaacgttgatacccaatgaaccattcgcatagctgccaaaatgcatccctcaagaccgttacggattgattacaggtgtctttagaacac
caactttatgagaaaacgaaaatcctaccagtcaaggaacacaatattacaatttctttagggttgacttcagtgaaatcaccccaaccccaacagttgtagtttcttcg
agttatatacatgatccacatcgttcattgcagtactgaacgatattcatagaggtacggtaaattgcagattatcgtattattatcgtacttggggaagaacacacacaca
cctattggtctcgtatagacagcacgactctcaaacacatgcccagaatgtgttgaagttcacaggctgaaagcatacaatttacggacaacgtaaagaggcagagatagc
ctcgccacggatcacataatcatgtaccgcactttgatctggcaaaaccatatagctcctgccgtatcggtatatatcggtatatatatatatttatatatattatcttccc
ctattccgaaatactcagtacggattccaacaaaataaagccaccttcatttacgcggaaatgatactgatattctttaaggaactaagtgaatttgtttaccttatttaaa
agtgatgtttattgacttccaaagaagcgaaaagaatcaatccatttattttaattgaaggtatattttttttgtactagaattttttgaaattaaacaaattttaatatta
tttgagtcaaattagttatattctataatggtacaatttcacttcctaataactgcaaaaaaagcaacataaaaattacttcctgagaatgacttcagatgtagtgaatttg
gaatcaaatataaaggacatcccttctatgtcaagcctgtgttaaaatcatcactttttttaggtctttttcagtacttccatggcgtcaattctacttctttttttgcttct
ttggaagttcttttttttgctgggtattgtcaattagaaagaccttcactttgtgcaacggcatacaaaagcttgtgcgttttcttggtatcggaataagcttctgtttttcc
agaaatctagaaatttttataccctacaccactatagtggggagggtattatacgtttgtgctgatgtttgtaacatacaaaaatattggtccaatacccaccttaaagtat
accgatcgtttcagaatcatttttttgagtcgattaagacatgtccgtccgtccgtctgtccggctggctgtccatgtaaaccttgtgcgcaaggtacaggccgcaattttc
aagataatttgacgaaatttggaccaagcatgttttttgacacagggacgaagcctattgaaaatggttgaaatcggtccattatttcacctagcccccatacaaccgtacc
tcccgatttgaacttttttatgccataattacgtcaaaaaattcagctatcacttttaaaaaattggcacaaataagtttttatataagtataaatgacactgcagattttcgtaag
gatcggcctatatttgaccctagccccccatacgaacccccccttcaaaaaatgacttaaacgtctaaaattgacttgtaaccatttgtatcgcaatgaaactcaacaaaacta
actgttatttagaaatatatcctttttcccaaattaaccaaggatcggtccatatttgacctatataaagcctcatttagaaattttagtttttttatcaataaattgcttaaa
tattttgaatttattggtaatattcaacataaaactttctttataaaaagtaaaacttttaaaaatatactcatggtgtagggtattatatggtcggccatgcccgactata
cttttctacttgtttctcttgttatgagggcaattttttagagccctcttagaaatatagccaaggcctgaagaatcataaaacttaaaaatttggtagtactttttaataaa
tattgactaaatttcatcttcttacaacccaatgtgcttccactaaatgttaaaaaatcagtttttccaaaacaacttttcttagaaatatttgcgaaaacaaaaacttttta
aaatatttcattcttgtaacaacattaacttggtaaatactatttgttatcaaaacatatacacacatgtacataactagtacatctacatacacaaatacacatcgacaaa
tacataagataaaatactaagtacttggaaattttgttatttacgagtattttcttataatattttcagGTTCTTGGTGCAGTTGTCACATACTTCATGGTGTTGGTGCAGC
TCAAGTAAaattatcaaatatacttacttaatgcaaaaacggcagcaactgcagcggcagctgcaaaaacatttgctgttgtatattttgcattggcaacatttttaagataa
gaaacagcaacaccagctacagcaacaataacactctgctaacaatgaaatataaaacaaggacattagcaacaacaacaacaaaaatatattttaaacttattttataaga
caacaaataattcacctacatttagaaacttttatacacacagatacatagtacttcaataccctatacttatatgtctgtgtgcatgtaaaagtattagtgtatctatgagc
tttctaataccaccacttatcaacttaatactaccattttatacagggcaaacccacatttttatacatttaagagttgtatggaaagttggttgtttgtttggttagttgcc
cttaaaggcaacaatagatgaagtaaatcagagaatttgatatattgctctcactttaaatttcatgtactggaatggctcaacaactgcatcaacagacgtttattttttgt
aaaaacatttattgtctaagggcgttggttgactgctgccatcatccacaggaatcatttagttgtcagttcttttctgctattttttgtttctcaaaaatatcttatattc
ttttagattttatcatttgctcgaacattattatttgttgttgtttttgttagaaaatatttctttagtttttaagtaatgttagagatttacattgaaaatgtaaacgaa
tatgtttcgtttaagtttttgaaaattactaaaaaattttgagaaaaatcgaatatttattattattttaaggtaaattcgcactcaatagaatttacgaaatttagaaaaa
ttacaaacattatttacaacttaataaagttcttattatttatgtttaagtatttaatacaaaattttatttaaaaaaattataaaaatttgttggaaataaataat

Figure S2: *LcupOrco* insert construction. (A) *LcupOrco* gene fragment including two sequences corresponding to the left (LHA) and right (RHA) homology arms of 1000 bp in length each one, necessary for the CRISPR homology directed repair (HDR) protocol cloned using plasmid pUCIDT-AMP GoldenGate (ordered from IDT). In addition, a DNA section including the restriction cutting sites (RCS) for enzymes XhoI (NEB) and NotI (NEB) was added in between of both

sequences in the same plasmid; **(B)** donor plasmid including the ZsGreen Marker followed by the *Hsp83* promoter located between positions 1891 to 7441 [2]; **(C)** ZsGreen Marker obtained from the donor plasmid. The plasmid was linearized using restriction enzymes BpmI (NEB), XhoI and NotI at 37°C for 60 min and the reaction was finished at 65°C for 20 min. After restriction digestion the mix was electrophoresed in a 1.5% agarose gel at 60 volts for 120 min. The band with the size corresponding to the marker (~5 Kb) was recovered from the gel and purified using the Zymoclean Gel DNA Recovery Kit (Zymo) following the manufacturer's specifications; **(D)** to obtain the final construct, 50 ng of linearized pUCIDT-AMP GoldenGate plasmid + 175.5 ng of the marker fragment (1:3 ratio) were ligated overnight (O/N) at 16°C using the T4 DNA Ligase (NEB). After ligation, 1 µl of the reaction mix was used to transform 10-beta competent *Escherichia coli* cells (NEB) following the manufacturer's specifications. Seventy-five µl of undiluted transformed 10-beta cells were poured into an Ampicillin 20 mg/ml (+Amp) Lysogeny Broth (LB) plate and incubated O/N at 37°C; **(E)** ten clones were recovered from the LB plate and grow in 3 mL of liquid LB +Amp O/N at 37°C and 250 rpm. To obtain the plasmids from clones the ZR Plasmid Miniprep Kit (Zymo) was used following the manufacturer's specifications and evaluated with the BpmI, XhoI and NotI restriction enzymes following the same protocols described above for restriction digestion and electrophoresis; **(F)** three positive plasmids for the selected restriction cutting sites were sequenced using Oxford Nanopore and aligned to the *in-silico* construct to confirm the sequence identity. Abbreviations: Kb = kilobase; MW = molecular weight.

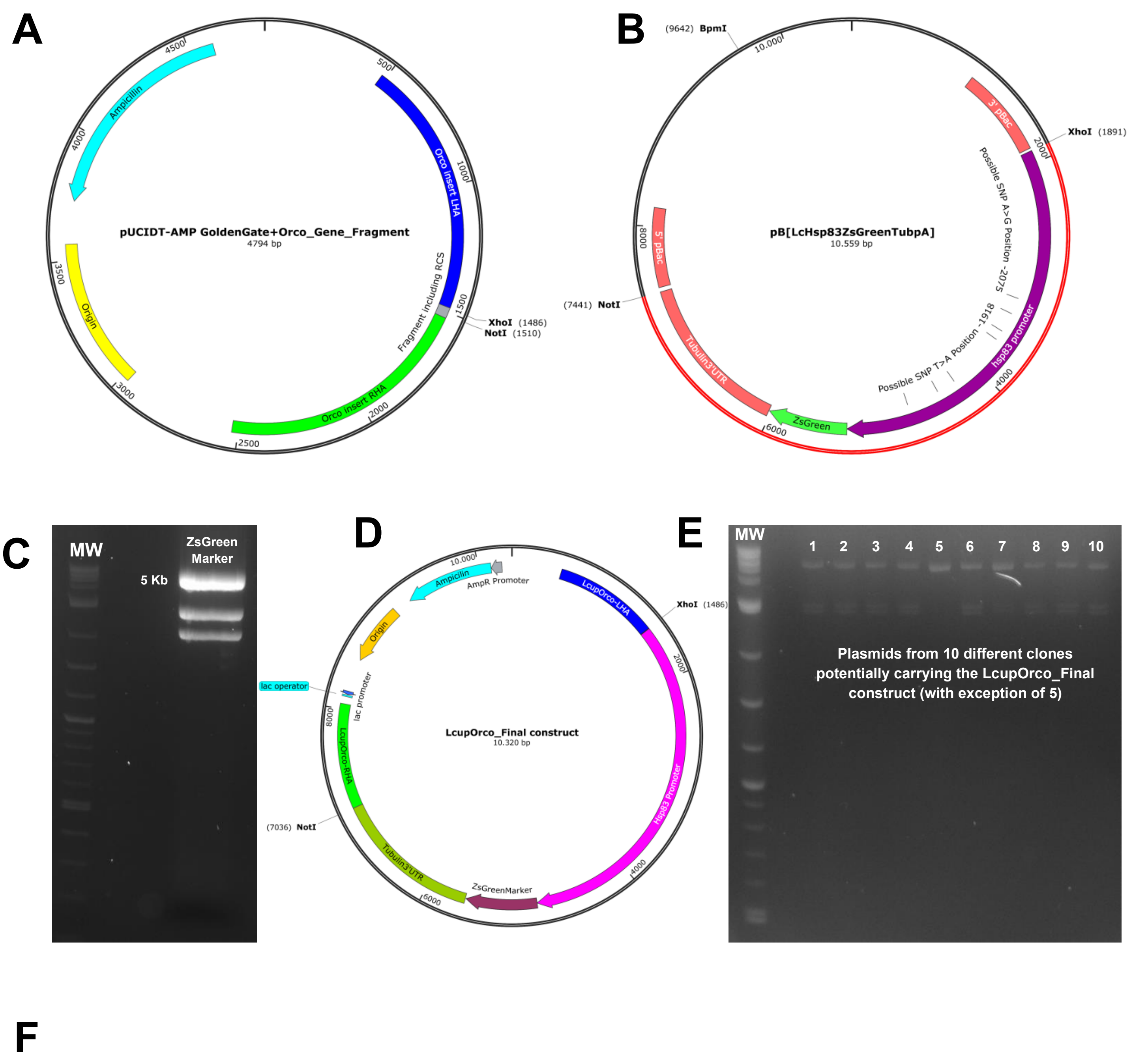

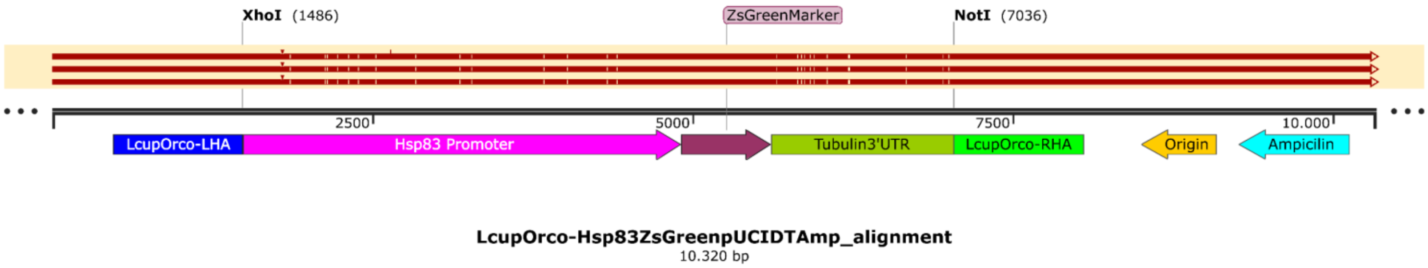

Figure S3: containers used for fly rearing and crosses. (A) plastic bottle used for fly rearing; **(B)** sandglass shaped arrangement made using two five ounces clear-plastic cups joined from removed bottoms used for fly crosses after embryo injections. These containers also included: a top holed cap and a bottom cap with an opening of ~ 1 inch in diameter; a rounded piece of white paper towel; a small glass vial filled with tap water and a small protein cookie made with yeast, milk and egg powder and cane sugar.

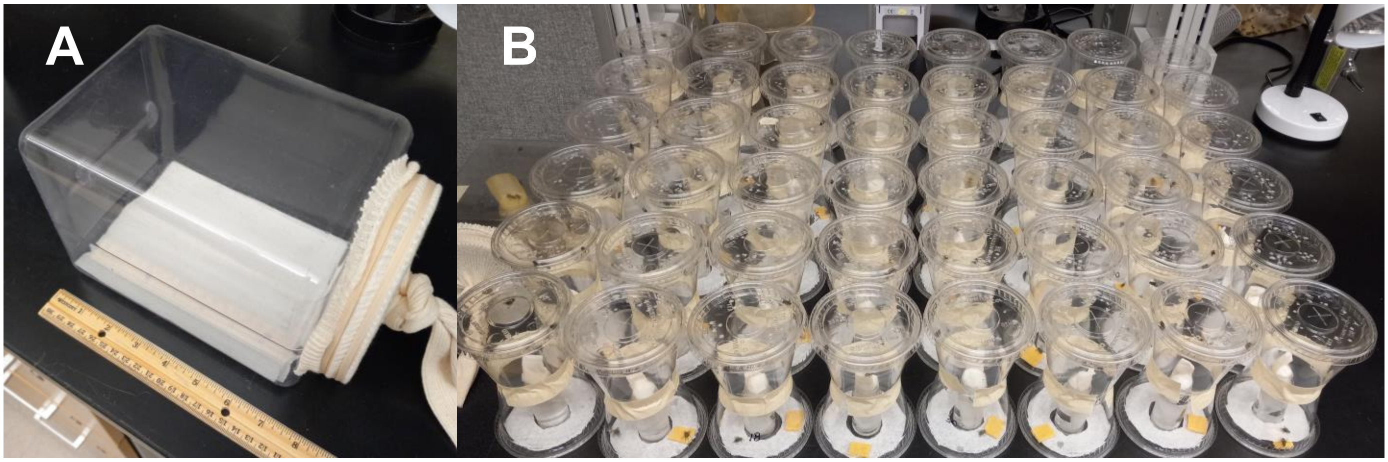

Figure S4: fly crosses completed after embryos microinjections. Abbreviations: G = generation; *wt* = wild-type.

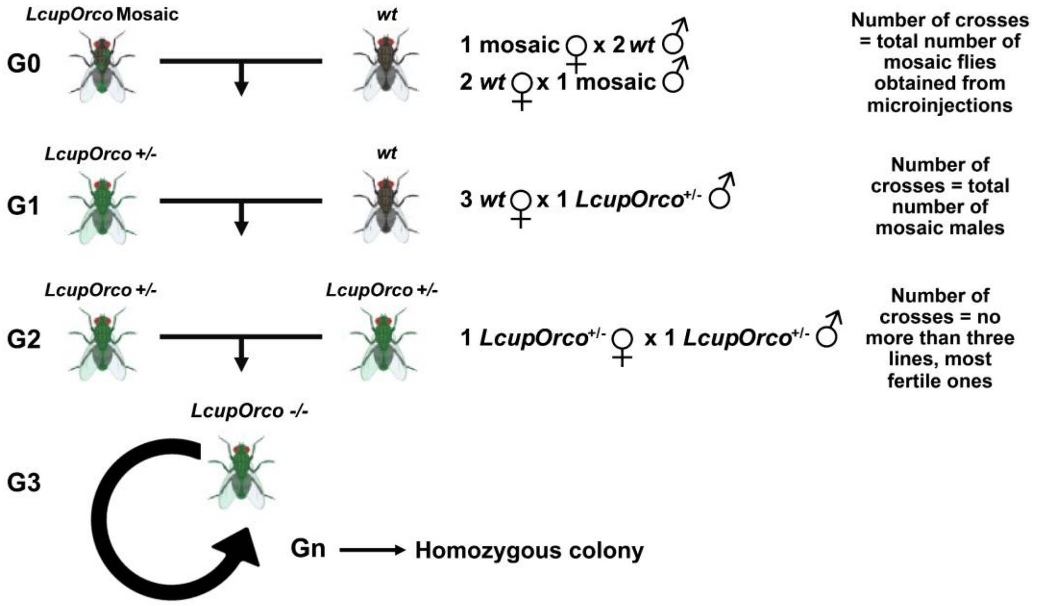

Note S2: primers used for *LcupOrco* insert detection of landing site. Three types of pair of primer associated with three different strategies were used to determine if the landing site of the *LcupOrco* insert was withing the *LcupOrco* locus in the *L. cuprina* genome. First one was associated with the left side (LS) of the insert including the upstream genome region, the *Hsp83* promoter and part of the green marker; the second one was designed to amplify the right side (RS) of the insert including the part of Tub3'-UTR and the downstream genome region; and third one was to amplify the whole *LcupOrco* insert (All) including from upstream to downstream *L. cuprina* genome regions flanking the *LcupOrco* locus.

Lcup-Orco-Genot-LS_Fw1: GCGAATACATTCTCCAAAGATTTCC
Lcup-Orco-Genot-LS_Rv1: GTTCAGTTCTAGTTCGGTTCTAGTT
Lcup-Orco-Genot-LS_Fw2: TGATTAGCGAATACATTCTCCAAAGATTTC
Lcup-Orco-Genot-LS_Rv2: GTTCGGTTCTAGTTCAGTTCTAGTTTAGT
Lcup-Orco-Genot-RS_Fw1: TGTATCATGGGACGCTAAGATCAA
Lcup-Orco-Genot-RS_Rv1: CTCTGACACAAACTTGGGTTCAAT
Lcup-Orco-Genot-RS_Fw2: TCTACCACGATGATCTCATTCACA
Lcup-Orco-Genot-RS_Rv2: TGCGTACCATCATTTCCTGTTTC
Lcup-Orco-Genot-RS_Fw3: TATTGTATCTACCACGATGATCTCATTCAC
Lcup-Orco-Genot-RS_Rv3: GCTACTTAGTTAGAATGGATGGAGTTAGTT
Lcup-Orco-Genot-All_FW1: ACTAGTTTAATGGAATGCGTTTCTC
Lcup-Orco-Genot-All_RV1: CTGACACAAACTTGGGTTCAATAA
Lcup-Orco-Genot-All_FW2: GCGAATACATTCTCCAAAGATTTCC
Lcup-Orco-Genot-All_RV2: AGTTAGTTGGGTCGCTAACTTATTT

PCR cycling parameters for LS and RS primers, using Q5 Taq (NEB)

Initial denaturalization = 3 min at 98°C.

Denaturalization = 30 sec at 98°C.
Annealing = 30 sec at 64°C. Repeat x 40 cycles
Extension = 2 min at 72°C.

Final extension = 5 min at 72°C.
Hold = ∞ at 10°C.

PCR cycling parameters for primers to amplify the whole insert, using LongAmp Taq (NEB)

Initial denaturalization = 3 min at 94°C.

Denaturalization = 30 sec at 94°C.
Annealing = 30 sec at 64°C. Repeat x 30 cycles
Extension = 7 min at 65°C.

Final extension = 10 min at 65°C.
Hold = ∞ at 10°C.

Reaction was performed in 20 µl of final volume and 300 nM for final primer concentration. For LS and RS amplification, the Q5 High-Fidelity Taq Pol was used, and for the whole insert amplification the LongAmp® Taq DNA Polymerase (NEB, Cat. # M0323S).

Supplementary Results

Table S1: RNA-Seq data overview. (A) each sample represents an RNA-Seq library. Sample's names, description and number of raw, trimmed and mapped reads per library are provided; **(B)** total sequences identified for the RNA-Seq experiment, divided by RNA type.

A

Sample	Description	Input (raw reads)	Seqs after trimming	Seqs that mapped to the reference genome	% of mapped reads
L1_1	Paired reads created from L1_1_R1 + L1_1_R2	52,830,504	52,205,318	48,807,734	93.5
L1_2	Paired reads created from L1_2_R1 + L1_2_R2	48,086,438	47,506,322	42,573,624	89.6
L1_3	Paired reads created from L1_3_R1 + L1_3_R2	42,877,238	42,330,090	37,676,550	89.0
L1_4	Paired reads created from L1_4_R1 + L1_4_R2	45,603,866	45,053,754	40,935,156	90.9
L1_5	Paired reads created from L1_5_R1 + L1_5_R2	41,328,256	40,821,682	37,217,254	91.2
L2_1	Paired reads created from L2_1_R1 + L2_1_R2	58,320,992	57,698,612	51,844,520	89.9
L2_2	Paired reads created from L2_2_R1 + L2_2_R2	41,450,914	40,964,162	36,594,770	89.3
L2_3	Paired reads created from L2_3_R1 + L2_3_R2	53,656,602	53,010,010	47,184,164	89.0
L2_4	Paired reads created from L2_4_R1 + L2_4_R2	42,815,302	42,325,130	38,215,588	90.3
L2_5	Paired reads created from L2_5_R1 + L2_5_R2	41,283,070	40,828,438	36,433,156	89.2
L3_1	Paired reads created from L3_1_R1 + L3_1_R2	49,724,376	49,257,306	43,834,497	89.0
L3_2	Paired reads created from L3_2_R1 + L3_2_R2	46,915,790	46,417,644	39,777,785	85.7
L3_3	Paired reads created from L3_3_R1 + L3_3_R2	47,115,312	46,591,974	42,029,106	90.2
L3_4	Paired reads created from L3_4_R1 + L3_4_R2	50,676,690	50,162,324	43,686,831	87.1
L3_5	Paired reads created from L3_5_R1 + L3_5_R2	48,385,720	47,878,954	41,611,198	86.9
L3_6	Paired reads created from L3_6_R1 + L3_6_R2	40,037,226	39,576,554	34,283,222	86.6
G1	Paired reads created from G1_R1 + G1_R2	376,305,232	363,255,256	330,378,422	90.9
G2	Paired reads created from G2_R1 + G2_R2	426,594,950	413,197,846	375,863,416	91.0
G3	Paired reads created from G3_R1 + G3_R2	389,811,044	376,623,516	342,584,417	91.0
G4	Paired reads created from G4_R1 + G4_R2	418,659,816	405,492,620	369,667,402	91.2
H1	Paired reads created from H1_R1 + H1_R2	368,202,822	356,051,540	327,903,784	92.1
H2	Paired reads created from H2_R1 + H2_R2	266,154,798	256,836,086	235,959,629	91.9
H3	Paired reads created from H3_R1 + H3_R2	421,364,048	405,731,648	373,815,153	92.1
H4	Paired reads created from H4_R1 + H4_R2	358,563,270	345,286,086	316,903,040	91.8
WL1	Paired reads created from WL1_R1 + WL1_R2	420,779,456	404,086,394	377,826,369	93.5
WL2	Paired reads created from WL2_R1 + WL2_R2	439,626,326	422,272,136	394,438,143	93.4
WL3	Paired reads created from WL3_R1 + WL3_R2	370,789,824	356,666,082	334,585,814	93.8

B

24207 sequences detailed as follows matched to the reference genome ID ASM2204524v1
- •21801 CDS (15547 annotated as genes)
- •1367 non-coding_RNA
- •420 misc_RNA (miscellaneous RNAs = small uncharacterized RNAs)
- •618 tRNA
- •1 uncharacterized RNA

Percent GC:

Sample	GC% before trimming	GC% after trimming
L1_1	39.8	39.6
L1_2	41.3	41.2
L1_3	41.1	40.9
L1_4	39.7	39.6
L1_5	39.6	39.4
L2_1	39.7	39.6
L2_2	40.4	40.3
L2_3	40.5	40.3
L2_4	38.5	38.3
L2_5	38.8	38.6
L3_1	40.5	40.4
L3_2	40.9	40.7
L3_3	41.3	41.1
L3_4	41.5	41.4
L3_5	41.2	40.9
L3_6	40.9	40.7
G1	36.7	36.5
G2	37.2	38.2
G3	34.4	37.2
G4	34.4	34.3
H1	40.6	40.5
H2	41.6	41.5
H3	41	41
H4	40.9	40.9
WL1	40.4	40.5
WL2	40.7	40.7
WL3	40	40.1

Figure S5: principal component analysis (PCA) completed using transcripts with a TPM expression ≥ 5 for all libraries (samples) associated with EXP-1 (larval stages). Samples WL1_1 to 3, WL2_1 to 3 and WL3_1 to 6 were collected from the LA07 colony. Samples WL1_4 and 5 and WL2_4 and 5 were collected from heterozygous individuals obtained from the crossing between the LA07 and the SLAM5X colonies. The PCA analysis did not show differences between samples of the same larval stage, collected from different colonies. Abbreviations: L1 = first larval state; L2, second larval stage; L3 = third larval stage; WL = whole larva sample; TPM = transcript per million.

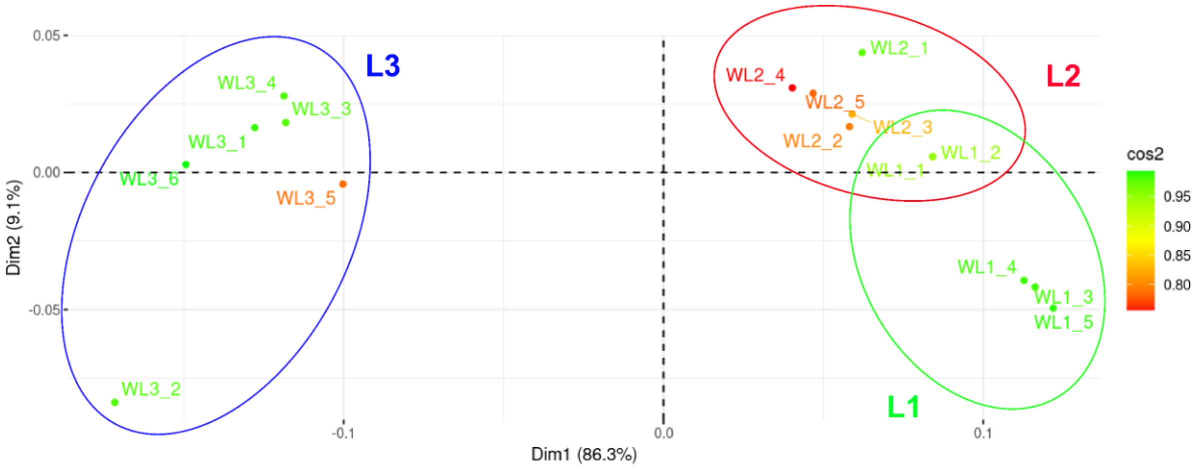

Figure S6: ABC transporters preliminary Neighbor Joining phylogenetic analysis. Abbreviations: Dmel = *Drosophila melanogaster*; Lcup = *Lucilia cuprina*; Lser = *Lucilia sericata*.

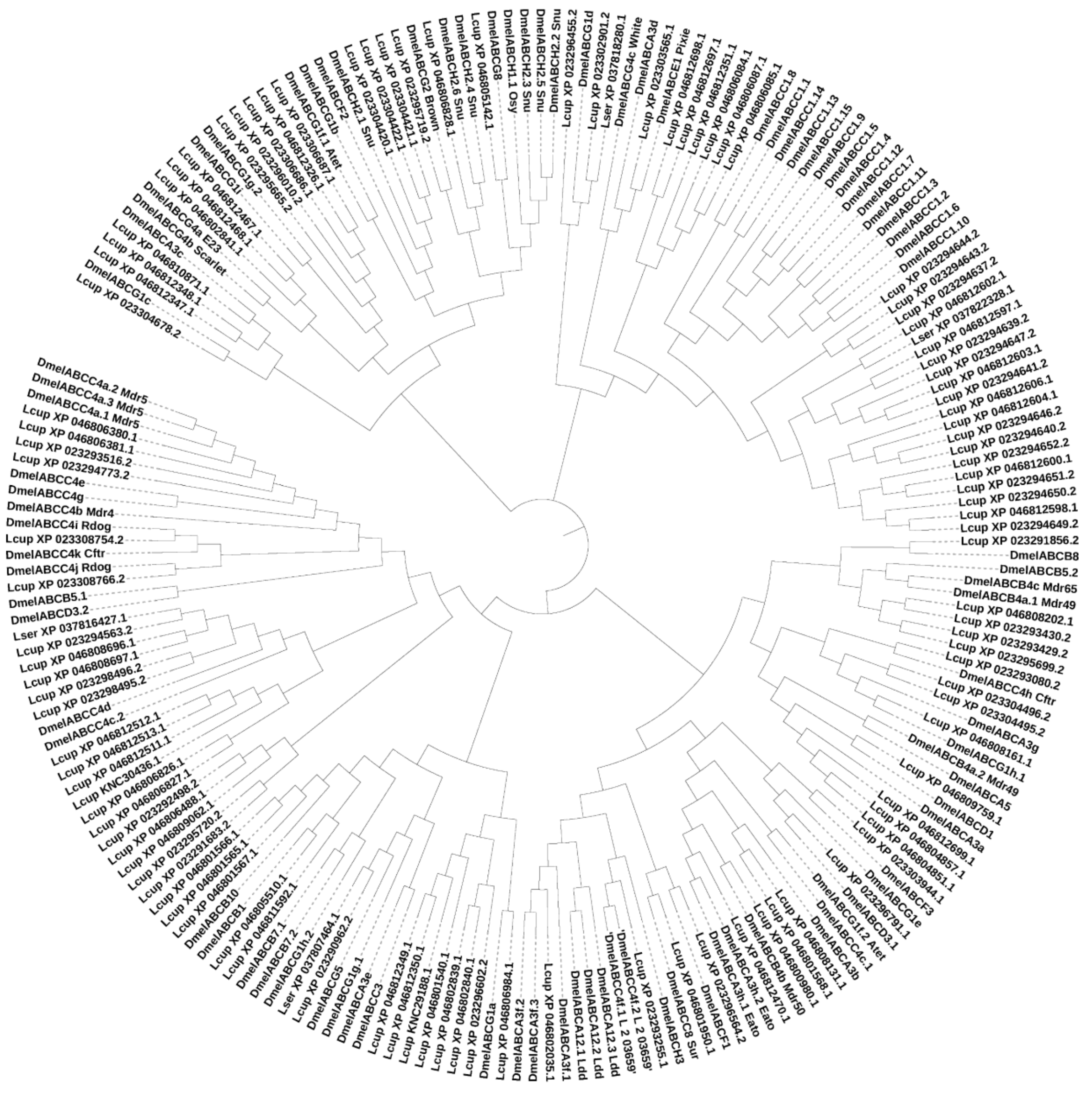

Figure S7: guide RNA (gRNA) cutting efficiency tested *in vitro* using the EnGen Spy Cas9. The amplicon size of *LcupOrco* gene fragment without cutting was 439 bp and after Cas9 *in vitro* cutting generated two overlapped amplicons of 224 and 215 bp, respectively. Abbreviations: C = control, uncut genomic DNA; MW = molecular weight; T = treated, gDNA + Cas9.

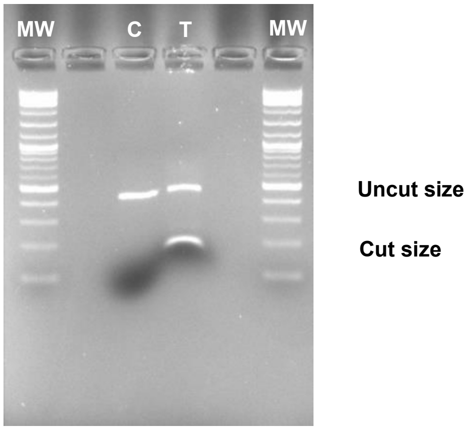

Figure S8: *Lucilia cuprina* eggs, larva stage 3 (L3) and adult flies expressing the ZsGreen marker before the genotyping analysis to determine *LcupOrco* insert landing site. (A) L3 showing transient expression of the marker 7 days after *wt* eggs injection; **(B)** eggs obtained from crossing a *LcupOrco* mosaic male with a *wt* female; **(C)** *wt* L3 on top vs. a heterozygous ZsGreen L3 at the bottom; **(D)** heterozygous ZsGreen L3 on top vs. homozygous for the same marker at the bottom; **(E)** dorsal view of a *wt* adult male (left) vs. G1 heterozygous ZsGreen male (right) under bright field; **(F)** same males showed in E under a green filter; ventral view of the same males using bright field **(G)** and a green filter **(H)**.

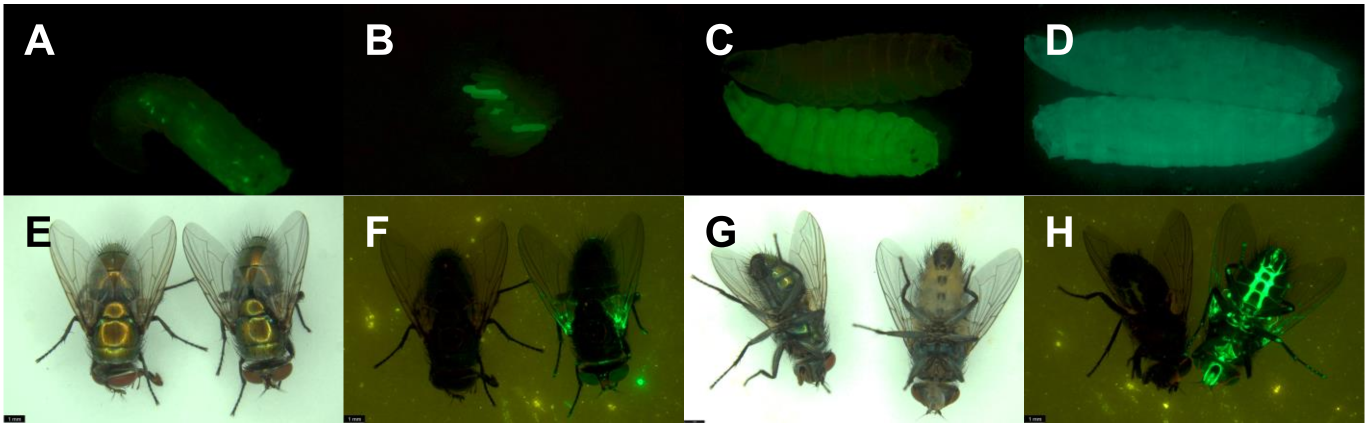

Figure S9: PCR amplification of gDNA of samples collected from *LcupOrco* larval diet preference test and female olfaction assay. (A) samples of larval olfaction assay; **(B)** samples of adult female olfaction assay. Primers, amplicon sequence and cycling parameters are detailed in **Note S1** (see Supplementary Materials and Methods section). Abbreviations: MW = molecular weight.

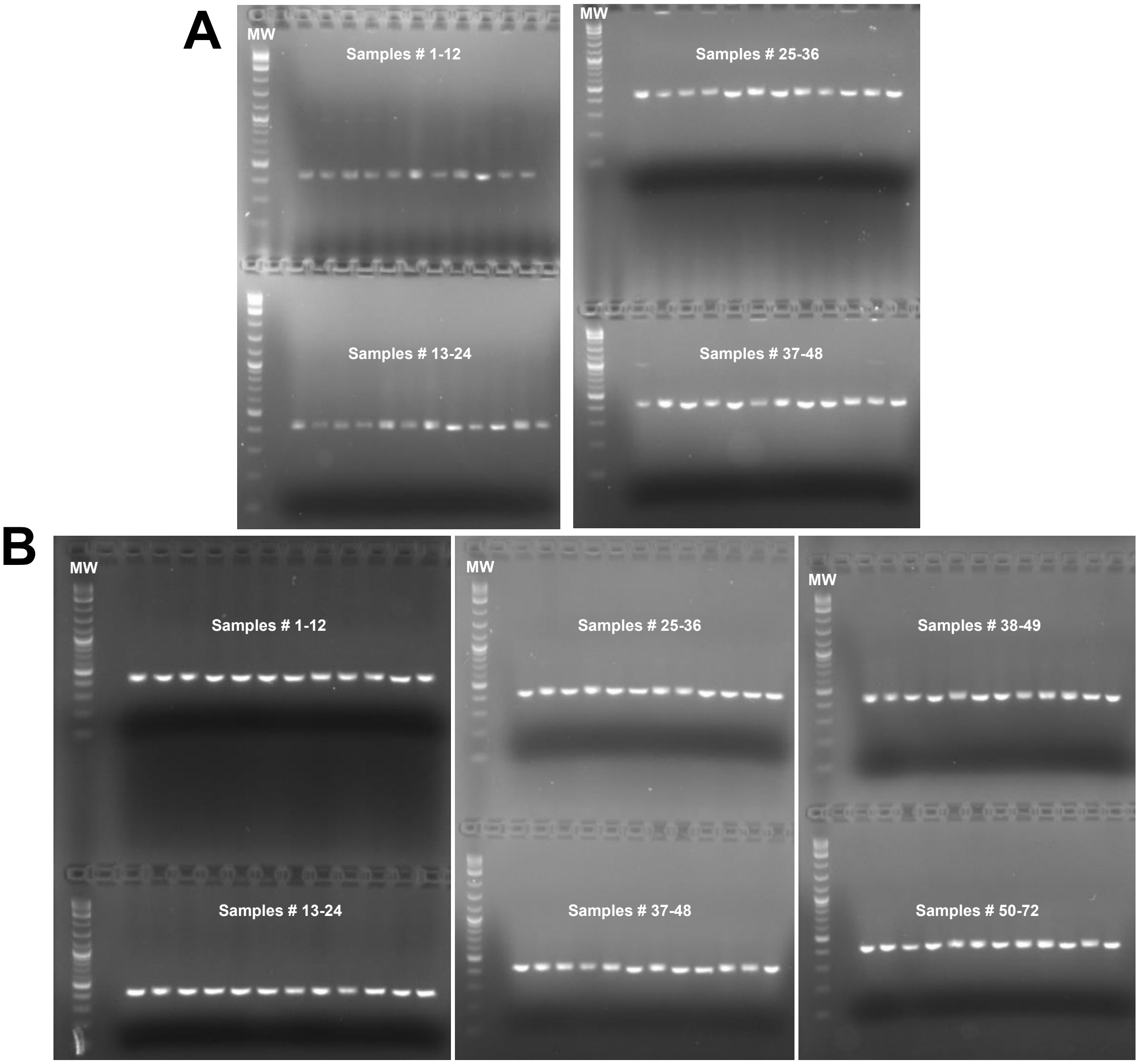

Figure S10: Sanger sequencing results for samples collected from *LcupOrco* larval diet preference test and adult female olfaction assay. (**A**) genotyped samples from #1 to 48 of larval diet preference test; and (**B**) genotyped samples from # 1 to 72 of adult female olfaction assay. Synthego software was used to analyze sample's chromatograms and indels % = 0 corresponds to *wt* samples, from 1 to 89% to heterozygous samples, and ≥ 90% to homozygous samples. Results were compiled along with single larva preferences in **Table S2 A-B** (see below).

A

Sanger Seq. Samples # 1-3

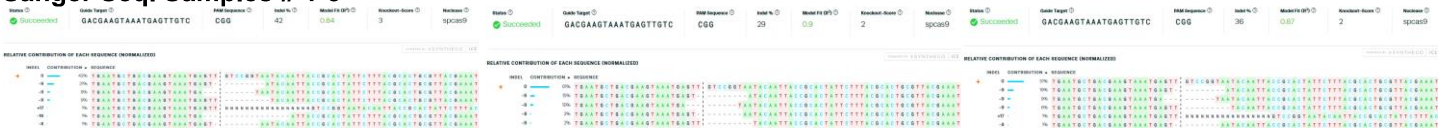

Sanger Seq. Samples # 4-6

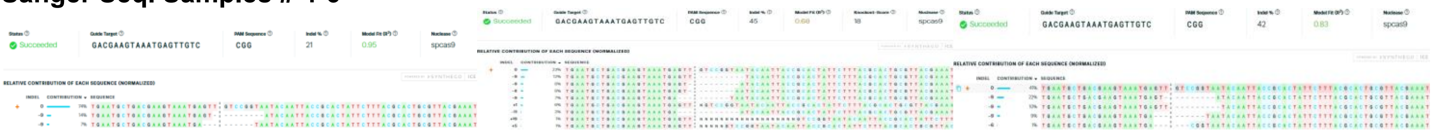

Sanger Seq. Samples # 7-9

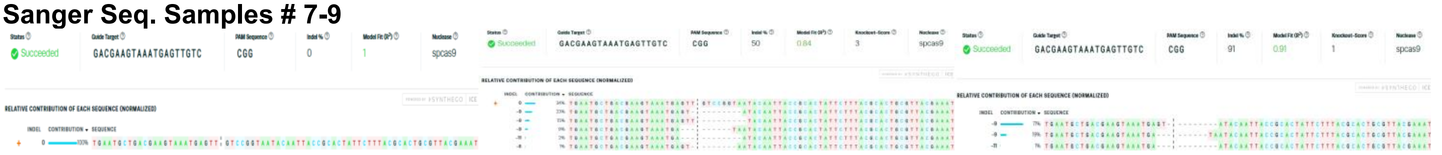

Sanger Seq. Samples # 10-12

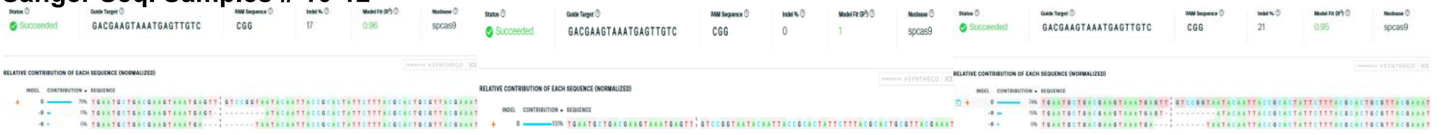

Sanger Seq. Samples # 13-15

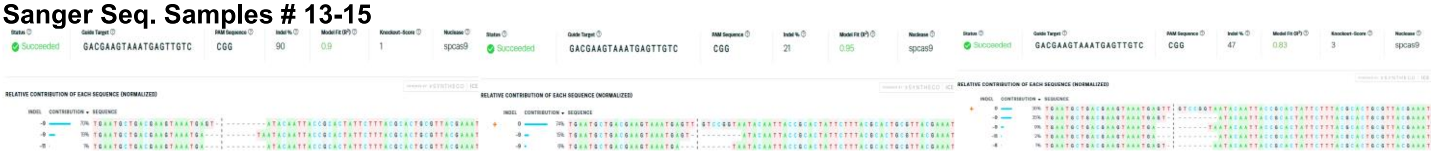

Sanger Seq. Samples # 16-18

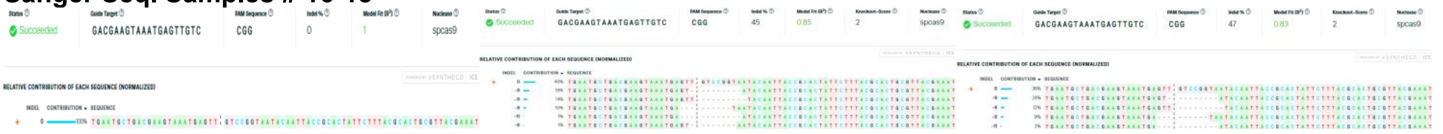

Sanger Seq. Samples # 19-21

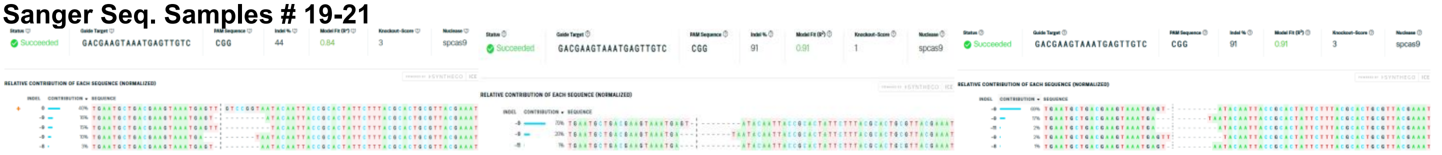

Sanger Seq. Samples # 22-24

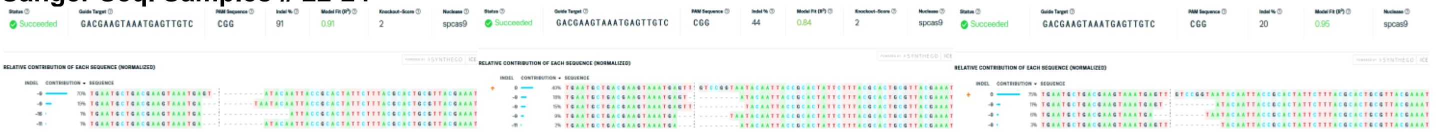

Sanger Seq. Samples # 25-27

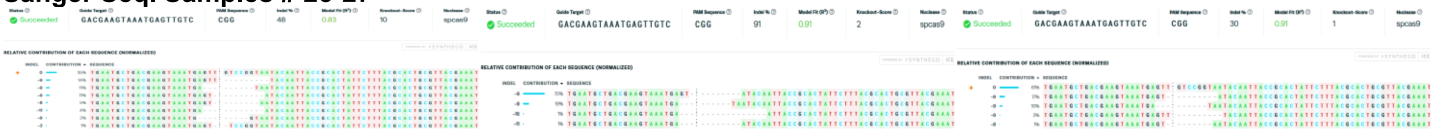

Sanger Seq. Samples # 28-30

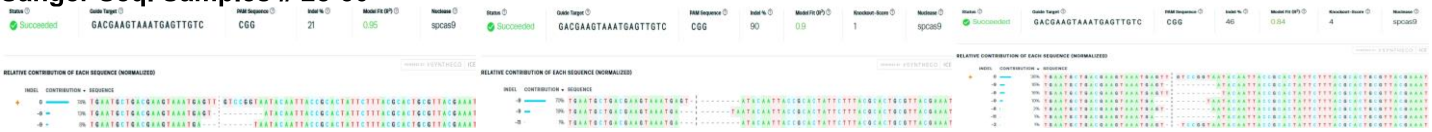

Sanger Seq. Samples # 31-33

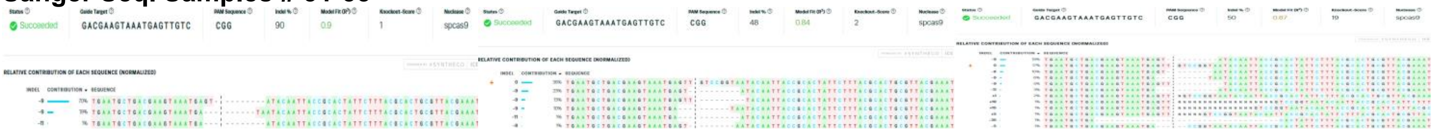

Sanger Seq. Samples # 34-36

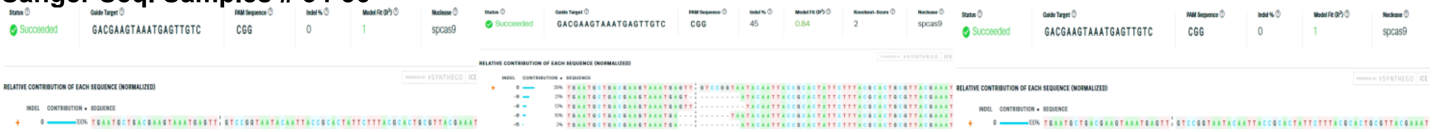

Sanger Seq. Samples # 37-39

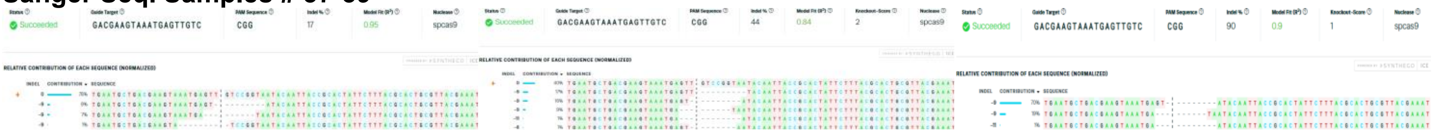

Sanger Seq. Samples # 40-42

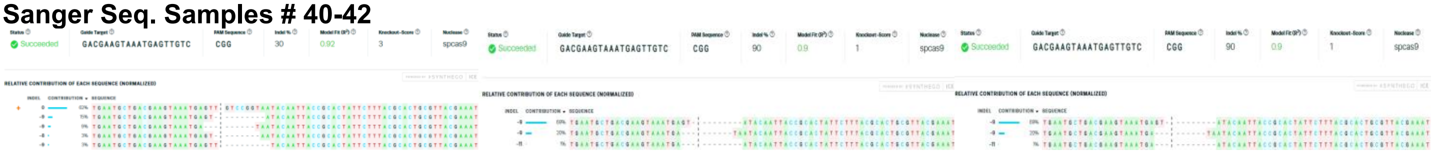

Sanger Seq. Samples # 43-45

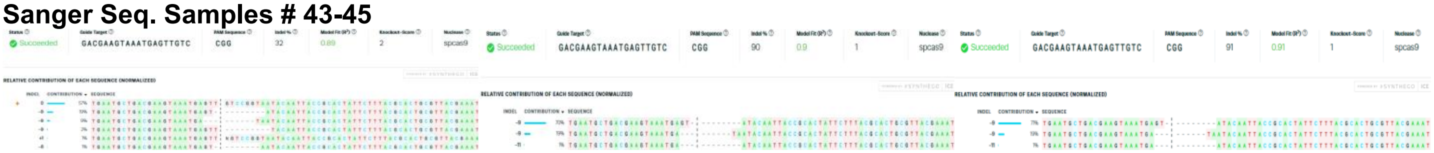

Sanger Seq. Samples # 46-48

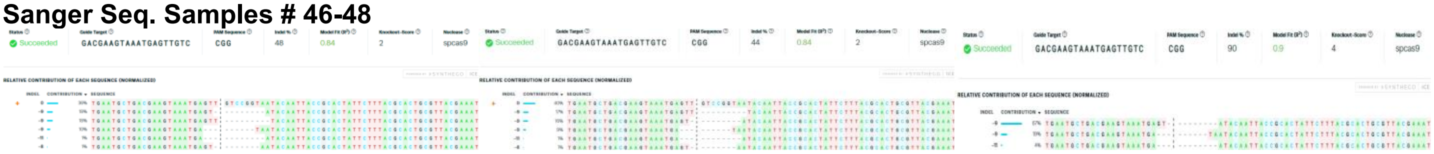

B

Sanger Seq. Samples # 1-3

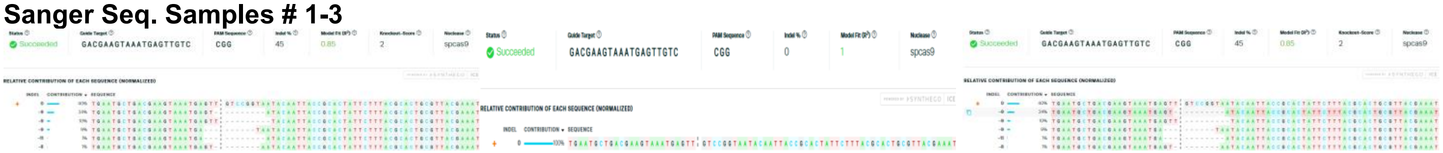

Sanger Seq. Samples # 4-6

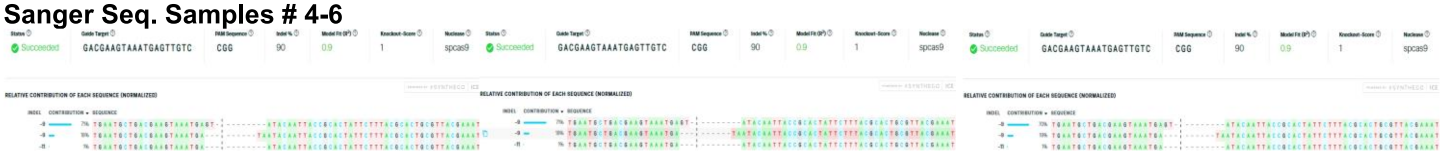

Sanger Seq. Samples # 7-9

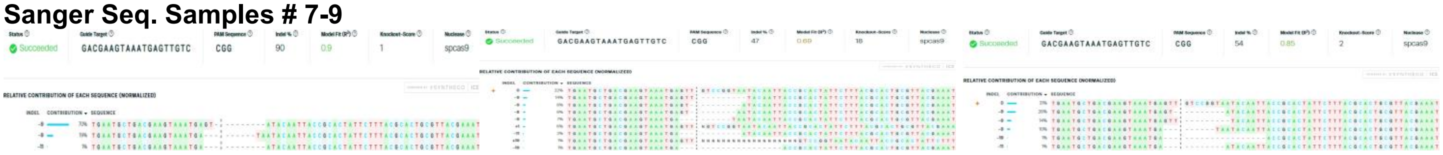

Sanger Seq. Samples # 10-12

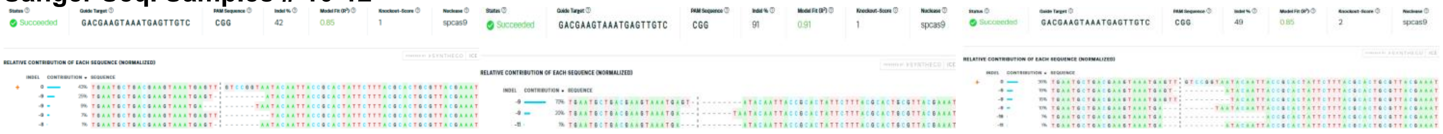

Sanger Seq. Samples # 13-15

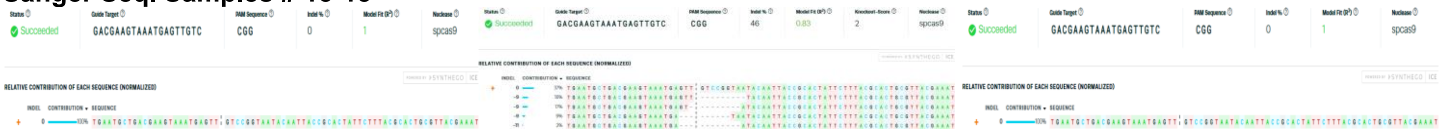

Sanger Seq. Samples # 16-18

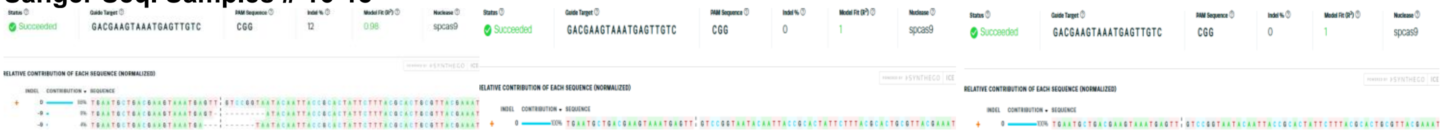

Sanger Seq. Samples # 19-21

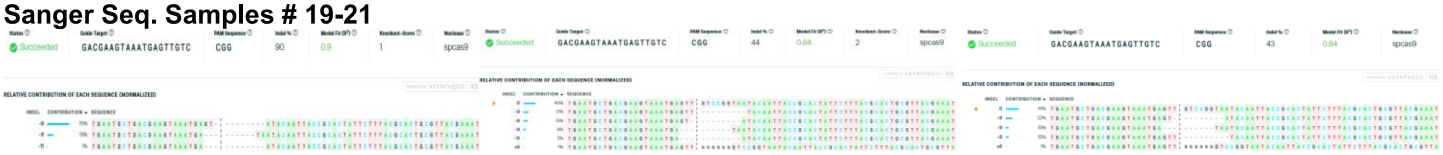

Sanger Seq. Samples # 22-24

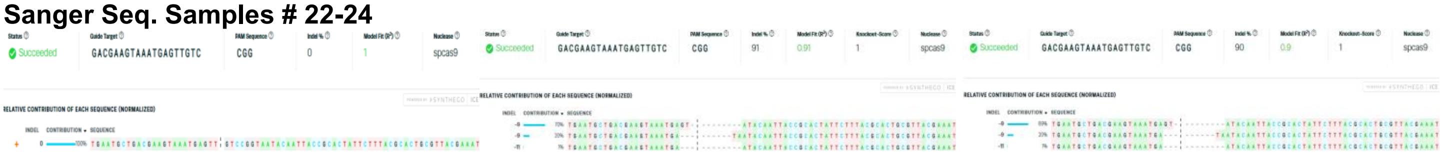

Sanger Seq. Samples # 25-27

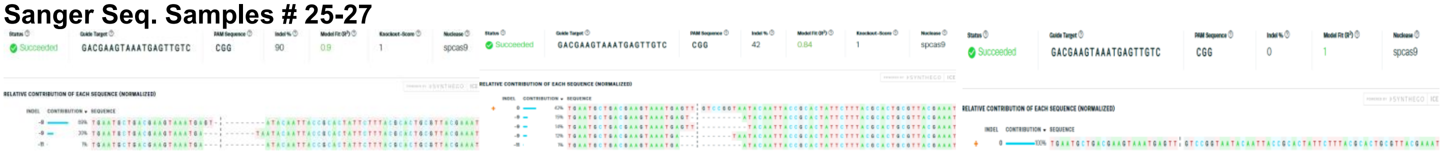

Sanger Seq. Samples # 28-30

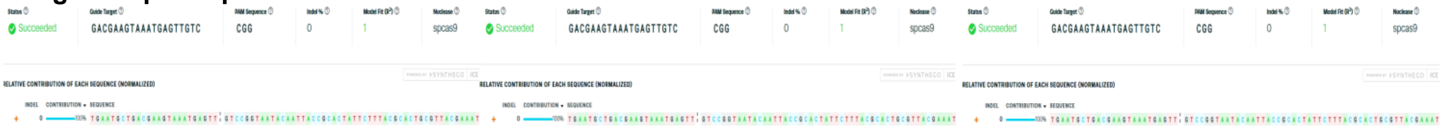

Sanger Seq. Samples # 31-33

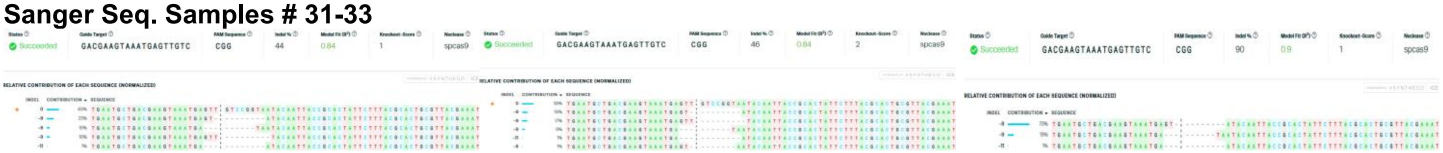

Sanger Seq. Samples # 34-36

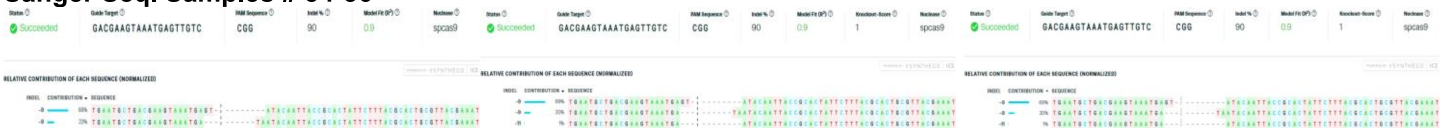

Sanger Seq. Samples # 37-39

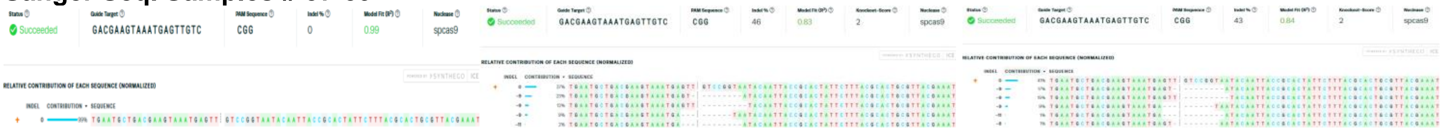

Sanger Seq. Samples # 40-42

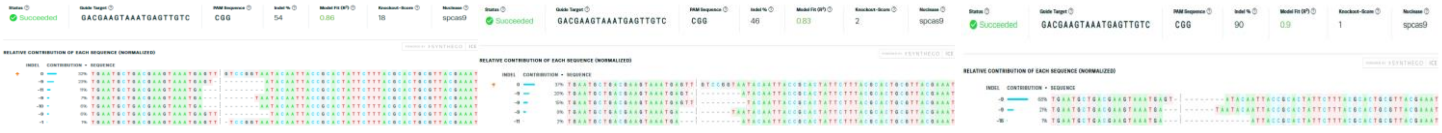

Sanger Seq. Samples # 43-45

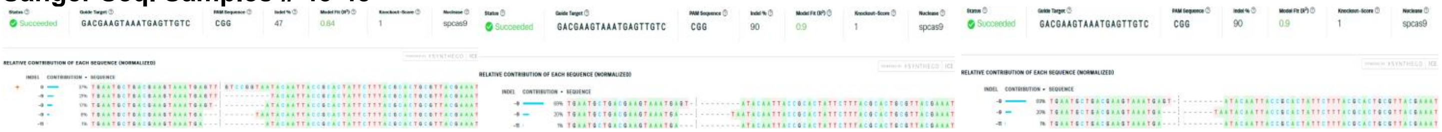

Sanger Seq. Samples # 46-48

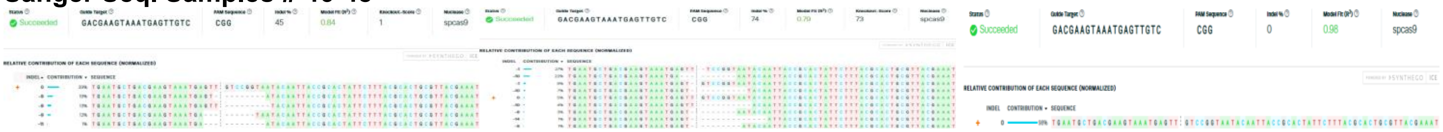

Sanger Seq. Samples # 49-51

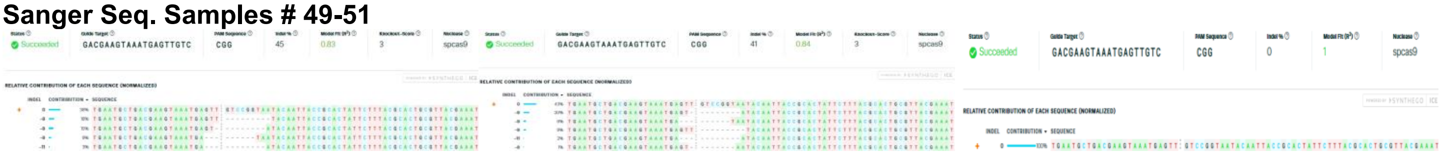

Sanger Seq. Samples # 52-54

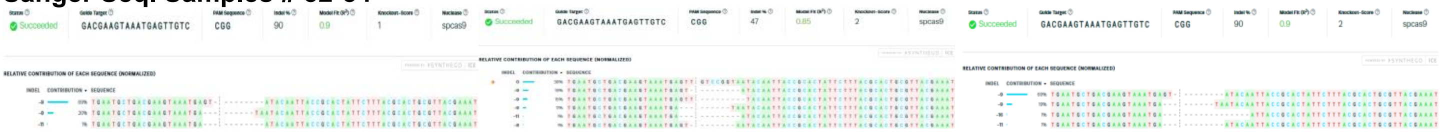

Sanger Seq. Samples # 55-57

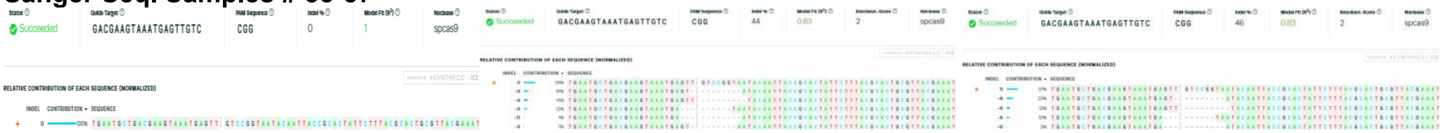

Sanger Seq. Samples # 58-60

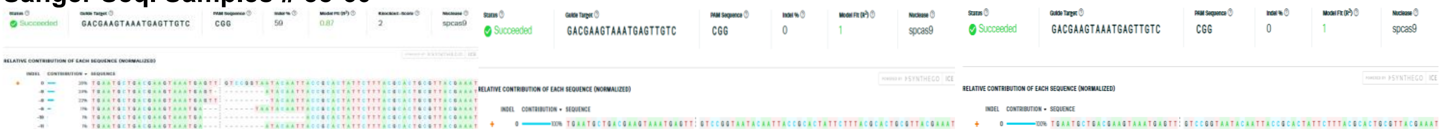

Sanger Seq. Samples # 61-63

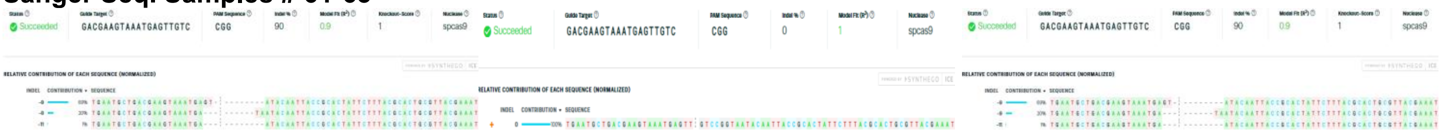

Sanger Seq. Samples # 64-66

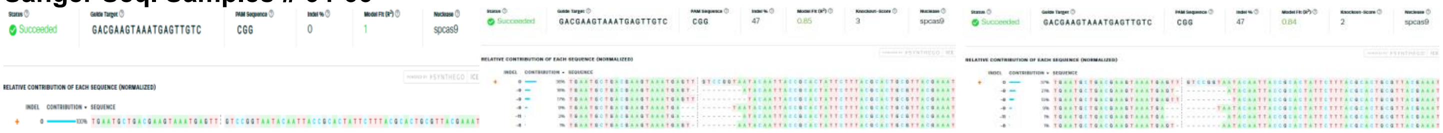

Sanger Seq. Samples # 67-69

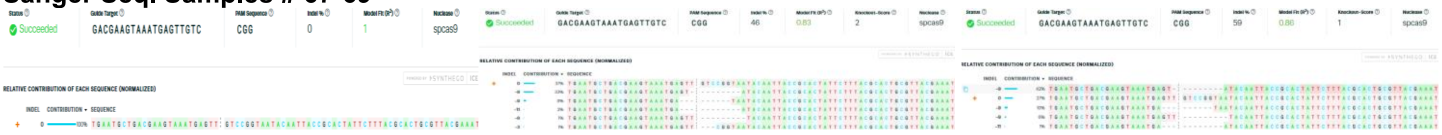

Sanger Seq. Samples # 70-72

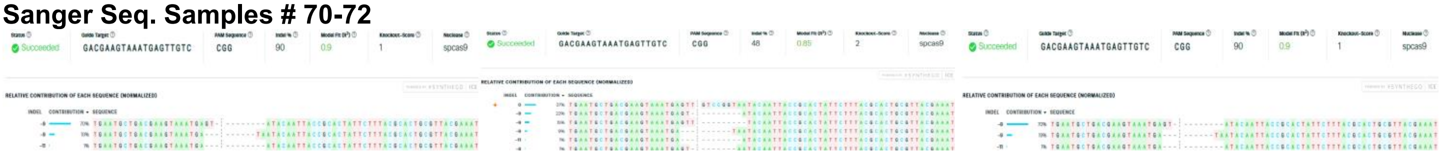

Table S2: compiled data of samples associated with *LcupOrco* behavioral assays. (A) larval diet preference test; **(B)** adult female olfaction assay. The numbers one through eight after the letters in table B, refer to the main chambers of the olfactometer and numbers after dots, to single females from the same chamber. Abbreviations: Fr = fresh beef (room temperature); FrCo = fresh cold beef; FrHo = fresh hot beef; HET = heterozygous; HOM = homozygous; NC = non-choice; NG = non-genotyped; Ro = rotten beef (room temperature); RoCo = rotten cold beef (25±1°C); RoHo = rotten hot beef (33±1°C); *wt* = wild-type. Hyphen symbol means non-tested by Sanger sequencing.

A

Larva #	Diet chosen	Larval weight	Sanger Seq. Sample #	Confirmed genotype by Sanger Seq.
**1**	RoCo	11.3	1	HET
**2**	RoCo	22	2	HET
**3**	RoCo	16.8	-	NG
**4**	RoCo	12.4	-	NG
**5**	RoCo	14.8	3	HET
**6**	RoHo	12.3	4	HET
**7**	FrCo	8	5	HET
**8**	FrCo	19.2	6	HET
**9**	RoCo	39.6	-	NG
**10**	RoCo	35.2	-	NG
**11**	FrCo	33.1	-	NG
**12**	RoCo	22.8	-	NG
**13**	RoCo	17.9	7	*wt*
**14**	RoCo	24	-	NG
**15**	RoCo	15.2	-	NG
**16**	RoHo	24.1	8	HET
**17**	RoCo	18.3	-	NG
**18**	RoCo	3.9	9	HOM
**19**	RoCo	16	-	NG
**20**	RoCo	11.9	10	HET
**21**	RoHo	6.8	-	NG
**22**	RoCo	32.6	-	NG
**23**	RoCo	17.1	-	NG
**24**	RoCo	14.1	11	*wt*
**25**	RoCo	29.7	-	NG
**26**	RoCo	20.8	-	NG
**27**	RoCo	14.7	12	HET
**28**	RoCo	15.2	-	NG
**29**	RoCo	3.8	-	NG
**30**	RoCo	19	-	NG
**31**	RoCo	9.8	13	HOM
**32**	RoCo	24.8	-	NG
**33**	RoCo	33.9	14	HET
**34**	RoCo	29.1	-	NG
**35**	RoCo	23.2	-	NG
**36**	FrCo	13.7	15	HET
**37**	RoHo	31	16	*wt*
**38**	RoCo	11.3	-	NG
**39**	RoCo	16.3	17	HET
**40**	RoCo	23.9	-	NG
**41**	RoCo	18.2	-	NG
**42**	RoCo	20	-	NG
**43**	RoCo	20.7	18	HET
**44**	RoCo	13.3	-	NG
**45**	RoCo	5	-	NG
**46**	RoHo	4.1	-	NG
**47**	RoHo	3	19	HET
**48**	RoHo	5.6	20	HOM
**49**	RoCo	19.5	-	NG
**50**	RoCo	18.8	21	HOM
**51**	RoCo	5.4	-	NG
**52**	RoCo	5.8	22	HOM
**53**	RoCo	21.2	-	NG
**54**	RoCo	14.7	23	HET
**55**	RoCo	20.6	24	HET
**56**	RoHo	31.4	-	NG
**57**	RoCo	10.8	-	NG
**58**	RoCo	10.2	25	HET
**59**	RoCo	21.7	-	NG
**60**	RoCo	13.6	26	HOM
**61**	FrCo	9.3	-	NG
**62**	RoCo	14.8	27	HET
**63**	RoCo	21.5	28	HET
**64**	RoCo	26.5	-	NG
**65**	RoCo	23	-	NG
**66**	RoHo	20	-	NG
**67**	RoHo	26.6	29	HOM
**68**	RoHo	15.4	-	NG
**69**	RoHo	16.5	30	HET
**70**	RoCo	16.7	31	HOM
**71**	RoCo	19.2	-	NG
**72**	RoCo	30.9	32	HET
**73**	RoCo	31.6	-	NG
**74**	RoCo	24.3	-	NG
**75**	RoCo	16.2	33	HET
**76**	FrCo	19.2	-	NG
**77**	FrCo	16.1	-	NG
**78**	RoHo	19	-	NG
**79**	RoCo	33.4	-	NG
**80**	RoCo	24.6	34	*wt*
**81**	FrCo	34.7	35	HET
**82**	RoHo	2.9	-	NG
**83**	RoHo	4.1	-	NG
**84**	RoHo	16.6	-	NG
**85**	RoCo	16.9	-	NG
**86**	RoCo	3.2	36	*wt*
**87**	FrCo	32.9	-	NG
**88**	RoHo	29.4	37	*wt*
**89**	RoHo	30.8	-	NG
**90**	RoCo	3.9	-	NG
**91**	FrHo	4.2	38	HET
**92**	RoHo	11.4	-	NG
**93**	RoHo	23.1	-	NG
**94**	RoCo	27.6	-	NG
**95**	RoCo	6.8	39	HOM
**96**	RoCo	10.6	-	NG
**97**	RoCo	22.5	40	HET
**98**	RoCo	32.2	-	NG
**99**	RoCo	3.8	41	HOM
**100**	RoCo	13.2	-	NG
**101**	NC	13.1	42	HOM
**102**	FrCo	25.7	43	HET
**103**	RoHo	20.9	44	HOM
**104**	RoCo	22.5	-	NG
**105**	RoCo	26.6	45	HOM
**106**	RoHo	29.5	-	NG
**107**	RoHo	6.7	-	NG
**108**	RoHo	18.3	46	HET
**109**	RoCo	9.8	-	NG
**110**	RoCo	8	-	NG
**111**	NC	4.6	47	HET
**112**	RoHo	9.9	-	NG
**113**	RoHo	4.5	-	NG
**114**	RoCo	12.2	48	HOM
**115**	RoCo	35.3	-	NG
**116**	RoCo	18.2	-	NG
**117**	RoCo	13.4	-	NG
**118**	RoCo	8.7	-	NG
**119**	RoCo	13.6	-	NG
**120**	RoCo	9.3	-	NG

B

Female #	Preference	Sanger Seq. Sample #	Confirmed genotype by Sanger Seq.
**1**	Ro1.1	1	HET
**2**	Ro1.2	2	*wt*
**3**	Ro1.3	3	HET
**4**	Ro1.4	NG	-
**5**	Ro1.5	NG	-
**6**	Ro1.6	NG	-
**7**	NC1.1	NG	-
**8**	NC1.2	4	HOM
**9**	NC1.3	NG	-
**10**	NC1.4	NG	-
**11**	NC1.5	NG	-
**12**	NC1.6	5	HOM
**13**	NC1.7	6	HOM
**14**	NC1.8	7	HOM
**15**	NC1.9	NG	-
**16**	NC1.10	NG	-
**17**	NC1.11	NG	-
**18**	NC1.12	NG	-
**19**	NC1.13	NG	-
**20**	NC1.14	NG	-
**21**	NC1.15	NG	-
**22**	Fr2.1	8	HET
**23**	Fr2.2	9	HET
**24**	Fr2.3	10	HET
**25**	Fr2.4	11	HOM
**26**	Ro2.1	12	HET
**27**	Ro2.2	13	*wt*
**28**	Ro2.3	14	HET
**29**	NC2.1	15	*wt*
**30**	NC2.2	16	HET
**31**	NC2.3	17	*wt*
**32**	NC2.4	NG	-
**33**	NC2.5	NG	-
**34**	NC2.6	NG	-
**35**	NC2.7	NG	-
**36**	NC2.8	NG	-
**37**	NC2.9	NG	-
**38**	NC2.10	NG	-
**39**	NC2.11	NG	-
**40**	NC2.12	NG	-
**41**	NC2.13	NG	-
**42**	NC2.14	NG	-
**43**	NC2.15	NG	-
**44**	NC2.16	NG	-
**45**	Fr3.1	18	*wt*
**46**	Fr3.2	19	HOM
**47**	Ro3.1	20	HET
**48**	Ro3.2	21	HET
**49**	Ro3.3	22	*wt*
**50**	Ro3.4	NG	-
**51**	Ro3.5	23	HOM
**52**	Ro3.6	NG	-
**53**	NC3.1	24	HOM
**54**	NC3.2	25	HOM
**55**	NC3.3	26	HET
**56**	NC3.4	NG	-
**57**	NC3.5	NG	-
**58**	NC3.6	NG	-
**59**	NC3.7	NG	-
**60**	NC3.8	NG	-
**61**	NC3.9	NG	-
**62**	NC3.10	NG	-
**63**	NC3.11	NG	-
**64**	Fr4.1	27	*wt*
**65**	Fr4.2	28	*wt*
**66**	Fr4.3	29	*wt*
**67**	Fr4.4	NG	-
**68**	Ro4.1	30	*wt*
**69**	Ro4.2	31	HET
**70**	Ro4.3	32	HET
**71**	Ro4.4	NG	-
**72**	NC4.1	33	HOM
**73**	NC4.2	34	HOM
**74**	NC4.3	35	HOM
**75**	NC4.4	NG	-
**76**	NC4.5	NG	-
**77**	NC4.6	36	HOM
**78**	NC4.7	NG	-
**79**	NC4.8	NG	-
**80**	NC4.9	NG	-
**81**	NC4.10	NG	-
**82**	NC4.11	NG	-
**83**	NC4.12	NG	-
**84**	NC4.13	NG	-
**85**	NC4.14	NG	-
**86**	NC4.15	NG	-
**87**	Fr5.1	37	*wt*

Female #	Preference	Sanger Seq. Sample #	Confirmed genotype by Sanger Seq.
**88**	Fr5.2	38	HET
**89**	Fr5.3	39	HET
**90**	Fr5.4	40	HET
**91**	Fr5.5	NG	-
**92**	Ro5.1	41	HET
**93**	NC5.1	42	HOM
**94**	NC5.2	NG	-
**95**	NC5.3	NG	-
**96**	NC5.4	NG	-
**97**	NC5.5	NG	-
**98**	NC5.6	NG	-
**99**	NC5.7	43	HET
**100**	NC5.8	44	HOM
**101**	NC5.9	45	HOM
**102**	NC5.10	NG	-
**103**	NC5.11	NG	-
**104**	NC5.12	NG	-
**105**	NC5.13	NG	-
**106**	NC5.14	NG	-
**107**	NC5.15	NG	-
**108**	NC5.16	NG	-
**109**	Fr6.1	46	HET
**110**	Fr6.2	47	HET
**111**	Fr6.3	48	*wt*
**112**	Fr6.4	NG	-
**113**	Ro6.1	49	HET
**114**	Ro6.2	50	HET
**115**	Ro6.3	51	*wt*
**116**	Ro6.4	NG	-
**117**	Ro6.5	NG	-
**118**	NC6.1	52	HOM
**119**	NC6.2	53	HET
**120**	NC6.3	NG	-
**121**	NC6.4	NG	-
**122**	NC6.5	54	HOM
**123**	NC6.6	NG	-
**124**	NC6.7	NG	-
**125**	NC6.8	NG	-
**126**	NC6.9	NG	-
**127**	NC6.10	NG	-
**128**	NC6.11	NG	-
**129**	NC6.12	NG	-
**130**	Fr7.1	55	*wt*
**131**	Fr7.2	56	HET
**132**	Fr7.3	57	HET
**133**	Ro7.1	58	HET
**134**	Ro7.2	NG	-
**135**	Ro7.3	59	*wt*
**136**	Ro7.4	60	*wt*
**137**	Ro7.5	NG	-
**138**	Ro.6	NG	-
**139**	Ro7.7	NG	-
**140**	Ro7.8	NG	-
**141**	Ro7.9	NG	-
**142**	Ro7.10	NG	-
**143**	NC7.1	61	HOM
**144**	NC7.2	62	*wt*
**145**	NC7.3	63	HOM
**146**	NC7.4	NG	-
**147**	NC7.5	NG	-
**148**	NC7.6	NG	-
**149**	NC7.7	NG	-
**150**	NC7.8	NG	-
**151**	NC7.9	NG	-
**152**	NC7.10	NG	-
**153**	Fr8.1	64	*wt*
**154**	Fr8.2	65	HET
**155**	Fr8.3	66	HET
**156**	Fr8.4	NG	-
**157**	Fr8.5	NG	-
**158**	Fr8.6	NG	-
**159**	Fr8.7	NG	-
**160**	Ro8.1	67	*wt*
**161**	Ro8.2	68	HET
**162**	Ro8.3	69	HET
**163**	Ro8.4	NG	-
**164**	NC8.1	70	HOM
**165**	NC8.2	71	HET
**166**	NC8.3	72	HOM
**167**	NC8.4	NG	-
**168**	NC8.5	NG	-
**169**	NC8.6	NG	-
**170**	NC8.7	NG	-
**171**	NC8.8	NG	-
**172**	NC8.9	NG	-
**173**	NC8.10	NG	-
**174**	NC8.11	NG	-

Figure S11: eggs obtained from *LcupOrco* mutated females. (A) eggs obtained from 8-day-old *LcupOrco*$^{+/-}$ females 3 days after crossing them to *NPF*$^{-/-}$ males expressing the ZsGreen marker. The same marker was removed from *LcupOrco* females after confirming that the *LcupOrco* insert landing site was not located withing the *LcupOrco* gene locus in the *L. cuprina* genome. The insert was removed by selecting non-fluorescent larvae and crossing males obtained from them for two generations vs. *wt* females. In addition, males from each generation were genotyped using protocols described in **Note S1** (see Supplementary Materials and Methods section) to confirm the presence of indels and point mutations within the *LcupOrco* coding region. The fluorescence in eggs confirmed the mating between the *LcupOrco*$^{+/-}$ females and *LcupNPF*$^{-/-}$ males and egg fertilization. *LcupOrco*$^{-/-}$ females did not lay eggs at this time; **(B)** part of *LcupOrco*$^{-/-}$ females laid eggs 10 days after mixing them with *LcupNPF*$^{-/-}$ males but eggs were not fertilized as showed under green filter **(C)**, and no larvae emerged from them; part of the *LcupOrco*$^{-/-}$ females laid fertilized eggs 17 days after mixing with *LcupNPF*$^{-/-}$ males, as showed under bright field **(D-F)** and green filter **(E-G)**; these eggs produced healthy progeny.

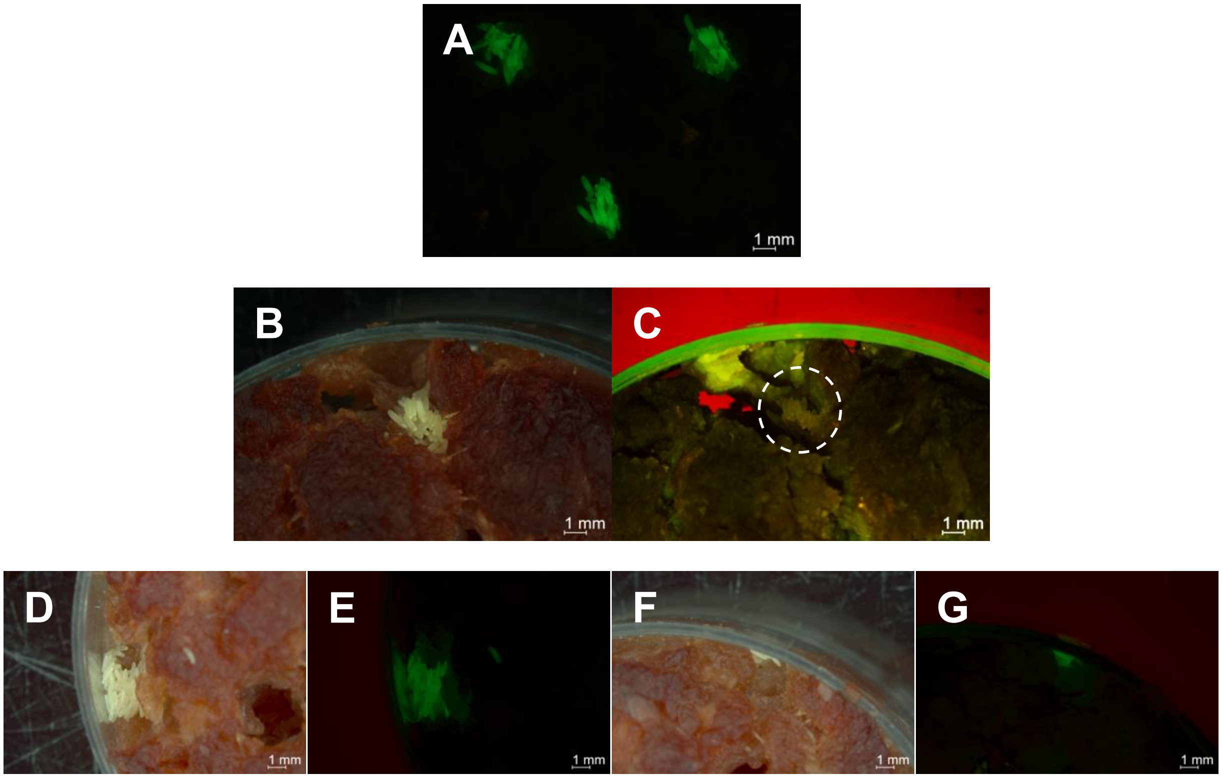

Figure S12: ovaries from 10-day-old *L. cuprina wt* and *LcupOrco* mutated females. Three females per condition were dissected (columns). All females were provided with tap water and sugar, but the presence of protein in the diet and mating condition changed between groups; *wt* virgin females not fed **(A-C)** and fed with protein **(D-F)**; *wt* mated females not fed **(G-I)** and fed with protein **(J-L)**; *LcupOrco*$^{-/-}$ mutated females fed with protein **(M-O)**.

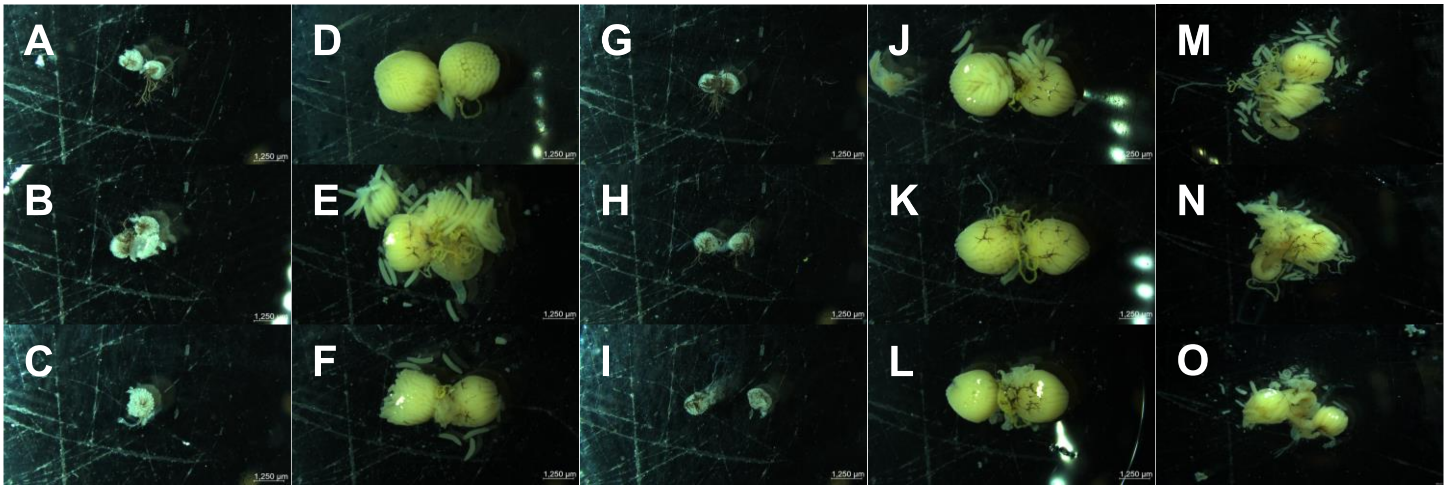

Supplementary References

1. Paulo, D. F. *et al.* Disruption of the odorant coreceptor Orco impairs foraging and host finding behaviors in the New World screwworm fly. *Sci Rep* **11**, 11379 (2021).
2. Concha, C. *et al.* Efficient germ-line transformation of the economically important pest species *Lucilia cuprina* and *Lucilia sericata* (Diptera, Calliphoridae). *Insect Biochem Mol Biol* **41**, 70–75 (2011).
